# Population Structure of Clinical *Vibrio parahaemolyticus* from 17 Coastal Countries, Determined through Multilocus Sequence Analysis

**DOI:** 10.1371/journal.pone.0107371

**Published:** 2014-09-16

**Authors:** Dongsheng Han, Hui Tang, Jun Lu, Guangzhou Wang, Lin Zhou, Lingfeng Min, Chongxu Han

**Affiliations:** 1 Clinical Medical Examination Center, Northern Jiangsu People’s Hospital, Yangzhou, Jiangsu, China; 2 Biobank Department, Northern Jiangsu People’s Hospital, Yangzhou, Jiangsu, China; 3 Department of Respiratory Medicine, Northern Jiangsu People’s Hospital, Yangzhou, Jiangsu, China; University of Ulster, United Kingdom

## Abstract

*Vibrio parahaemolyticus* is a leading cause of food-borne gastroenteritis worldwide. Although this bacterium has been the subject of much research, the population structure of clinical strains from worldwide collections remains largely undescribed, and the recorded outbreaks of *V. parahaemolyticus* gastroenteritis highlight the need for the subtyping of this species. We present a broad phylogenetic analysis of 490 clinical *V. parahaemolyticus* isolates from 17 coastal countries through multilocus sequence analysis (MLST). The 490 tested isolates fell into 161 sequence types (STs). The eBURST algorithm revealed that the 161 clinically relevant STs belonged to 8 clonal complexes, 11 doublets, and 94 singletons, showing a high level of genetic diversity. CC3 was found to be a global epidemic clone of *V. parahaemolyticus*, and ST-3 was the only ST with an international distribution. *recA* was observed to be evolving more rapidly, exhibiting the highest degree of nucleotide diversity (0.028) and the largest number of polymorphic nucleotide sites (177). We also found that the high variability of *recA* was an important cause of differences between the results of the eBURST and ME tree analyses, suggesting that *recA* has a much greater influence on the apparent evolutionary classification of *V. parahaemolyticus* based on the current MLST scheme. In conclusion, it is evident that a high degree of genetic diversity within the *V. parahaemolyticus* population and multiple sequence types are contributing to the burden of disease around the world. MLST, with a fully extractable database, is a powerful system for analysis of the clonal relationships of strains at a global scale. With the addition of more strains, the pubMLST database will provide more detailed and accurate information, which will be conducive to our future research on the population structure of *V. parahaemolyticus*.

## Introduction

*Vibrio parahaemolyticus* is a halophilic bacterium inhabiting marine and estuarine environments. Since it was first isolated in Japan in 1950 as a cause of food-borne illness, *V. parahaemolyticus* has been recognized as an important seafood-borne pathogen responsible for acute diarrheal illness in humans [1]. Consumption of raw or undercooked seafood is the primary route of *V. parahaemolyticus* infection [2]. The organism possesses a number of virulence factors, including thermostable direct hemolysin (TDH) [3], thermostable direct hemolysin-related hemolysin (TRH) [4], and type 3 secretion system 1 (TTSS1) and TTSS2 [5]. Strains that possess *tdh*, *trh*, and TTSS2-encoding genes are generally pathogenic and are responsible for the vast majority of clinical cases, while such strains account for few isolates collected from various marine species [6], [7], [8].

The public health and commercial burdens associated with *V. parahaemolyticus* contamination around the world are high. In the United States, approximately 34,664 (18,260–58,027) cases of food-borne illness are caused by *V. parahaemolyticus* each year, leading to a mortality rate of 0.9% [9]. In many Southeast Asian countries, such as Japan, Taiwan, and Vietnam, approximately half of food-poisoning outbreaks are caused by this pathogen [10], [11]. Therefore, ongoing monitoring of *V. parahaemolyticus* is important for preventing infections and guiding future intervention strategies. With the development of molecular biology methods, many molecular typing techniques have been widely applied in molecular epidemiological studies of *V. parahaemolyticus* [12], [13], [14]. These tools have played an important role in analyses of the genetic background of *V. parahaemolyticus* as well as in tracking the source of infections and investigating the route of transmission. Gonzalez-Escalona et al. [13] developed a successful MLST scheme for *V. parahaemolyticus* in a study on 100 strains of global origin and demonstrated that it was a powerful genetic fingerprinting technique with a higher resolution for molecular epidemiological and population genetic studies of this pathogen. Subsequent studies utilized MLST to successfully examine the genetic diversity of global isolate collections [15] as well as geographically restricted populations [16], [17], [18], [19]. Several MLST analyses further confirmed that *V. parahaemolyticus* undergoes frequent recombination and shows an epidemic population structure [15], [16].

In the present study, we took full advantage of the PubMLST database (http://pubmlst.org/vparahaemolyticus/) to investigate the clonal and phylogenetic relatedness of clinical *V. parahaemolyticus* isolates in a more global context. A total of 490 clinical strains with sequence type (ST) information were ultimately included in our analyses. These isolates were both temporally (collected from 1951 to February 2014) and geographically (collected from 17 countries distributed in Asia, the United States, South America, Africa and Europe) diverse. We sought to elucidate the population structure of clinical *V. parahaemolyticus* strains spread throughout the world population and to examine the potential application of MLST to molecular epidemiological studies of this pathogen. We provide a depiction of the global distribution of the genetic population structure of this pathogen, and we predict that the results of this study will inform future efforts to detect pathogenic strains and prevent disease outbreaks.

## Materials and Methods

### Data on bacterial strains

As of February 2014, data on a total of 522 clinical *V. parahaemolyticus* isolates were available in the pubMLST database, among which 490 isolates with sequence type (ST) information were included in this analysis (see Table S1). The isolates were temporally (collected from 1951 to February 2014) and geographically (collected from 17 countries distributed in Asia, the United States, South America, Africa and Europe) diverse.

### Assignment to clonal complexes

The assignment of STs to clonal complexes was accomplished using eBURST V3 (http://eburst/mlst.net) as described previously [20], [21]. The statistical confidence in the ancestral types was assessed using 1,000 bootstrap resamplings. Inclusion in a clonal complex was restricted to STs sharing all 7 alleles as well as single-locus variants (SLVs), which share at least 6 of the 7 alleles [22]. Two STs are considered to be single-locus variants (SLVs) when they differed from each other at a single locus. Double-locus variants (DLVs) are defined as any two STs differing at two loci. For each clonal complex, eBURST identifies the ST that is most likely to represent the founding genotype (the primary founder). The primary founder is predicted on the basis of parsimony as the ST with the largest number of SLVs in the clonal complex. Singletons are defined as STs differing at two or more alleles from every other ST [22].

### Phylogenetic analysis

Minimum-evolution (ME) trees for each locus and for the concatenated sequences of each ST were constructed with Mega 4 software using the Kimura two-parameter model to estimate genetic distances. The statistical support of the nodes in the ME tree was assessed through 1,000 bootstrap resamplings.

### Locus statistics

To evaluate the potential for the loci used in our typing schemes to be subject to varying degrees of selection, we calculated the number of alleles, number of polymorphic sites and nucleotide diversity per site using DnaSP V5 [23]. The ratio of nonsynonymous-to-synonymous substitutions (d*N*/d*S*) was calculated via the Nei and Gojobori method, as implemented in START V2 [24]. This statistic is a measure of selection, where d*N*/d*S*<1 indicates purifying selection; d*N*/d*S* = 1 indicates neutral selection; and d*N*/d*S*>1 indicates positive selection.

## Results

### Nucleotide diversity at each locus

The nucleotide sequence data for the seven housekeeping gene fragments for the 490 *V. parahaemolyticus* isolates are summarized in Table 1. The number of alleles observed for each locus ranged from 57 (*pntA*) to 86 (*gyrB*), and the percentage of polymorphic nucleotide sites varied from 10.40% (44) (*tnaA*) to 24.28% (177) (*recA*). The nucleotide diversity ranged from 0.012 to 0.028, with the highest degree of diversity being observed for *recA* (0.028). The most frequently occurring alleles at each locus were *dnaE3 (17), gyrB4 (27), recA19 (23), dtdS4 (20), pntA29 (23), pyrC4 (17)* and *tnaA22 (22)*. The d*N*/d*S* ratio was lower than 1 for each locus (Table 1), which indicates that nonsynonymous sites are evolving more slowly than synonymous sites and suggests that these genes have mainly been affected by purifying selection during their evolution.

**Table 1:** Genetic Diversity of the Seven Loci in 490 *Vibrio parahaemolyticus* Isolates.

Locus	Fragmentsize (bp)	No. of alleles	Nucleotidediversity(per site)	No. ofpolymorphicsites	% Variablesites	d*_N_*/d*_S_*
Chromosome I:						
*dnaE*	557	70	0.014	64	11.49	0.024
*gyrB*	592	86	0.025	64	10.81	0.001
*recA*	729	79	0.028	177	24.28	0.015
Chromosome II:						
*dtdS*	458	74	0.026	50	10.92	0.002
*pntA*	430	57	0.012	45	10.47	0.029
*pyrC*	493	72	0.013	52	10.55	0.057
*tnaA*	423	59	0.012	44	10.40	0.018

### STs generated via MLST and identification of Clonal Complexes

**Figure 1:**
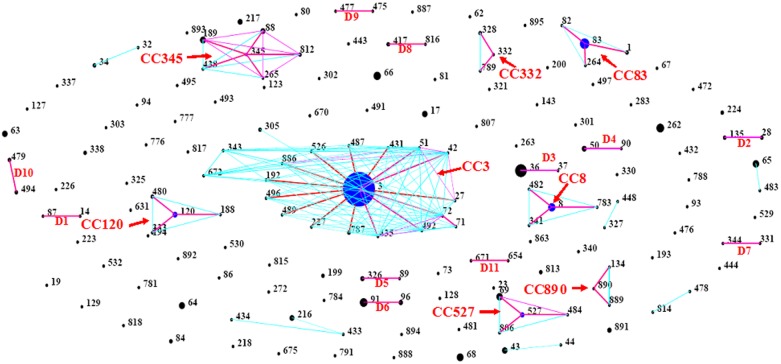
V. parahaemolyticus “population snapshot” obtained using eBURST v3. Eight clonal complexes (CC3, CC345, CC83, CC8, C120, CC527, CC332 and CC890), 11 doublets (D1 to D11), and 94 singletons were identified. STs that are SLVs of each other are connected by red lines. DLV STs are connected by blue lines. The sizes of the circles are relative to the number of strains in the ST.

A total of 161 STs were identified among the 490 isolates, indicating a high degree of genotypic diversity (see Table S1). Among these STs, 120 contained single isolates, while 40 STs included between 2 and 23 isolates, and ST3 was most frequent (179 out of 490 isolates). The application of eBURST to our data resolved the 161 STs into 8 clonal complexes (CC3, CC345, CC83, CC8, C120, CC527, CC332 and CC890), 11 doublets (D1 to D11), and 94 singletons (Fig. 1). CC3 remained the most populated clonal complex, being comprised of 196 strains with 17 different STs. ST-3 was defined by eBURST as the ancestral type for CC3. The 17 STs of CC3 differed at the *dnaE* (5 variants), *gyrB* (5 variants), *recA* (4 variants), *pyrC* (4 variants) and *tnaA* loci (2 variants) (see Table S2). Additionally, they shared identical allele sequences of *dtdS* and *pntA,* which revealed *dtdS4 e* and *pntA29* to be two highly conserved gene sequences during the evolution of CC3. The other 7 CCs included between 3 and 6 STs, which were mostly recovered in Asia and America.

### Geographical distribution of the STs

The geographical distributions of the different STs are shown in Table 2. ST-3 was isolated on all five continents. China was the country where the most strains were uploaded, with as many as 113 STs being found in this country. The USA, Thailand and Japan were additional countries where *Vibrio parahaemolyticus* subtypes were widely distributed.

**Table 2:** Geographic distribution of the 490 *Vibrio parahaemolyticus* Isolates.

Continent/Country		No. of strains	No. of STs	Mainly STs
Asia	China	189	113	ST3, ST8, ST69, ST120, ST216, etc.
	Thailand	40	16	ST3, ST262, ST91, ST83, ST17, ST8, etc.
	Japan	32	16	ST3, ST83, ST1, ST8, ST84, ST91, ST189, etc.
	India	27	6	ST3, ST8, ST83, ST189, ST217, ST326
	Bangladesh	15	4	ST3, ST14, ST51, ST65
	Korea	3	3	ST3, ST27, ST217
	Singapore	5	1	ST3
	Indonesia	1	1	ST3
	Philippines	1	1	ST8
	Maldives	2	1	ST96
North America	USA	58	19	ST3, ST36, ST43, ST50, ST65, ST135, ST417, etc.
South America	Peru	50	8	ST3, ST19, ST64, ST65, ST88, ST89, ST93, ST94
	Chile	28	5	ST3, ST28, ST63, ST64, ST65
	Ecuador	2	2	ST3, ST71
Europe	Norway	5	5	ST3, ST34, ST73, ST80, ST81
	Spain	3	2	ST3, ST17
Africa	Mozambique	29	5	ST3, ST66, ST67, ST68, ST69

### Phylogenetic analysis

**Figure 2:**
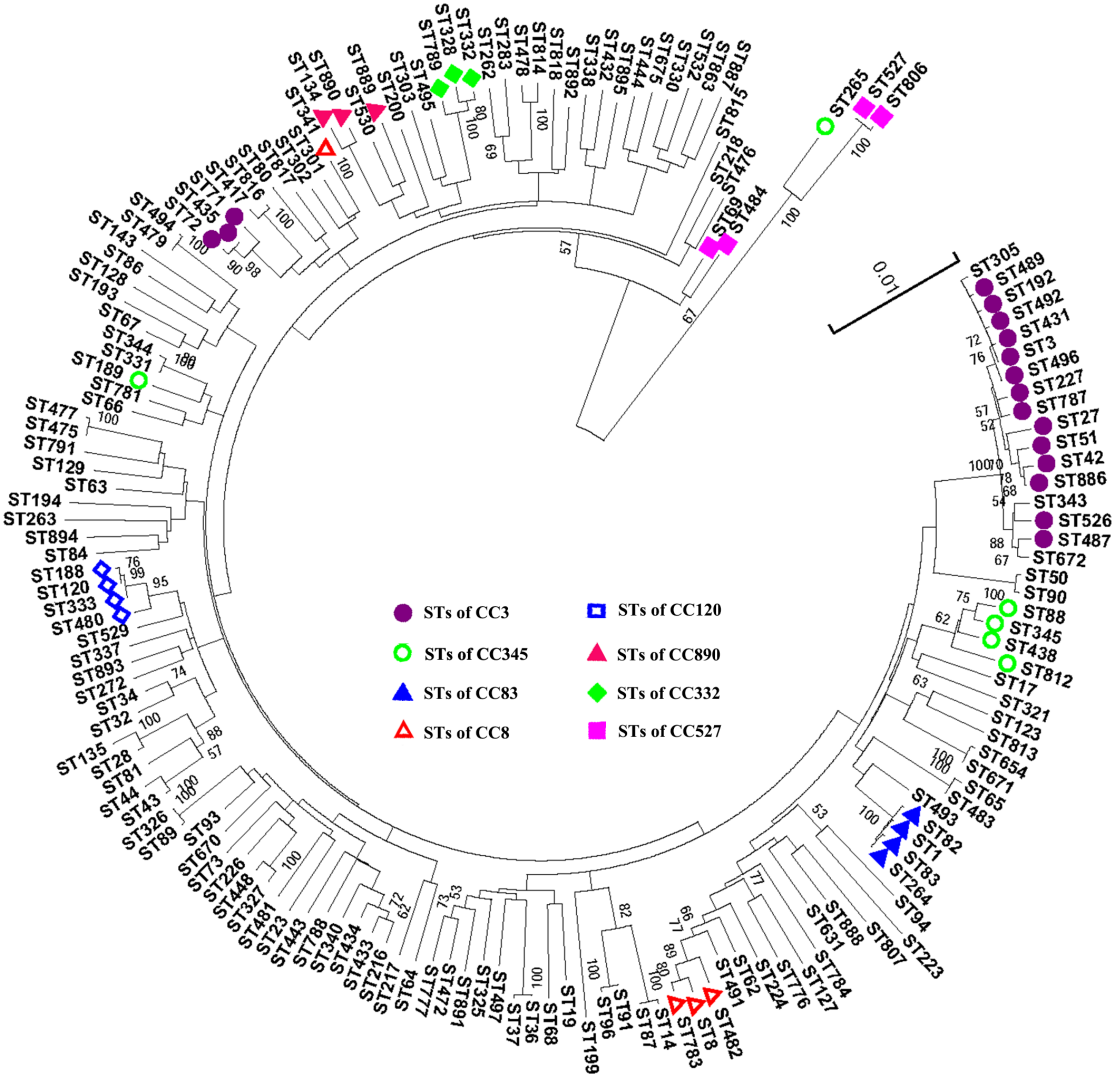
An ME tree was constructed using the concatenated sequences of the seven loci of each of the 161 STs obtained in this study. Squares, circles, and triangles with different shading represent the eight clonal complexes observed via eBURST. The scale represents the evolutionary distance, and bootstrap values over 50% are shown on the branches.

An ME tree representing the concatenated sequences of the seven housekeeping gene fragments in the 490 isolates is shown in Fig. 2. Generally, the eBURST and ME tree analyses were consistent, but the ME tree provided a better resolution and elucidated some phylogenetic relationships among CCs or singletons that were not observed or resolved by eBURST. CC83, CC120, and CC890 identified by eBURST formed individual clusters in the ME tree (Fig. 2). The 11 different doublets also grouped together in the ME tree. In the ME tree, CC3 was divided into two branches, one of which was comprised of ST71, ST72 and ST435, while the other was comprised of an additional 13 STs. A distant evolutionary relationship was observed between the two branches, an three singletons (ST305, ST343 and ST672, three DLVs of ST3) exhibited a relatively closer evolutionary distance to CC3 than ST71, ST72 and ST435 in the ME tree analysis. We speculate that the highly degree of nucleotide diversity of the housekeeping gene *recA* (average 0.028) was the main reason for the distant evolutionary relationship observed between ST71, ST72 and ST435 (ST71 (*recA19→recA4*), ST72 (*recA19→recA4*) and ST435 (*recA19→recA31*) and the other members of CC3. We found that *recA* gene nucleotide diversity was not present in ST305, ST343 and ST672 (compared with the ancestral type ST3) (see Table S2). In fact ST71, ST72 and ST435 showed differences of 25, 25, and 23 SNPs from the common *recA* allele found in CC3 members (*recA19*), whereas ST305, ST343 and ST672 only differs from ST3 by 2 SNPs (1 in *dtdS* and 1 in *pntA*), from ST3 by 9 SNPs (*recA*), and from ST3 by 11 SNPs (2 in *dnaE* and 9 in *gyrB*). Similarly, the diversity of *recA* had an important impact on the classification of other CCs. For example, the ME tree showed that ST189 (*recA19→recA3*) was not closely related to CC345; ST341 (*recA82→recA127*) might not belong to CC8; and CC527 was divided into two branches. Most of the isolates belonging to the clonal complexes differed only at the *recA* locus and were indistinguishable at the other six loci analyzed. Therefore, we believe that the high degree of nucleotide diversity in *recA* plays an important role in the phylogenetic analysis of *V. parahaemolyticus.*

## Discussion

An MLST scheme has been applied to a geographically diverse panel of *V. parahaemolyticus* strains to investigate their population structure [13], [25]. The application of the MLST scheme to sufficient numbers of *V. parahaemolyticus* strains in different laboratories would facilitate determination of the global distribution of the epidemiology and genetic population structure of this pathogen. In this study, we present an MLST-based analysis of the *V. parahaemolyticus* population structure among 490 clinical isolates collected in different laboratories in 17 countries since 1951. We provide a worldwide depiction of the epidemiology and extent of diversity of this pathogen, which is spreading in the world population. It would appear that multiple sequence types are contributing to human infection. The high degree of genetic diversity within *V. parahaemolyticus* populations might present a significant threat to human health and warrants continued monitoring.

The low d*N*/d*S* ratio obtained for all seven genes suggests that purifying selection has been dominant for these housekeeping genes. This finding was consistent with the conclusions of Narjol González-Escalona et al. [13] and Jeffrey W. Turner et al. [21]. A total of 161 different allelic combinations and an average of 71 alleles per locus were identified, indicating a high degree of genotypic diversity at slowly evolving loci. Among the seven genes, *recA* presents the highest degree of nucleotide diversity (0.028) and the greatest number of polymorphic nucleotide sites (177). Previous MLST studies have confirmed that recombination is evident as a significant source of genetic diversity in *V. parahaemolyticus* populations [13], [15], [16], [18]. Highly divergent *recA* alleles and evidence of frequent recombination at this locus were also observed in previous studies [25], [26], [27]. According to our analysis of *V. parahaemolyticus* isolates and the findings of previous studies, *recA* has a major influence on the apparent phylogenetic relationships and population structure of *V. parahaemolyticus* obtained based on the current MLST scheme.

As many as 161 sequence types were identified among the 490 clinical isolates, demonstrating that the *V. parahaemolyticus* population is extremely diverse. ST3 was the most frequent sequence type, in agreement with the findings of other investigators [28], [29], [30]. In the present study, ST-3 was found to be the sole sequence type with an international distribution. In China, the survey revealed that approximately 74.3% and 94.0% of *V. parahaemolyticus* infections were caused by ST3 in patients in Beijing [31] and Zhejiang [29], respectively, and 85.4% of O3:K6 serotype isolates belonged to ST3 in Shenzhen [30]. All of these results showed that ST3 plays an important role in human infection. The eBURST algorithm revealed that the 161 clinically relevant STs belonged to 8 clonal complexes, 11 doublets, and 94 singletons. Strains of CC3 first emerged in India in 1996 [32] and then in other Asian countries [33], on the American continent [33], in Europe [34], [35], and even in Africa [36], and CC3 ultimately became a global epidemic clone of *V. parahaemolyticus*, posing a significant public health threat.

The clustering, eBURST and ME tree analyses were largely consistent with one another. However, the ME tree provided a better resolution and elucidated some phylogenetic relationships among clonal complexes or singletons that were not observed using eBURST. Three clonal complexes (CC83, CC120, and CC890) and all eleven doublets identified by eBURST formed individual clusters in the ME tree, showing that they were genetically exclusive groups, while the strains of CC3, CC345, CC8 and CC527 were divided into different clusters in the ME tree. Further analysis showed that most of the clonal complex isolates differed only at the *recA* locus and were indistinguishable at the other six loci analyzed. The variability in the *recA* gene suggests that this gene has evolved more rapidly and played a much greater role in the evolutionary classification of the *V. parahaemolyticus* population.

In the present study, we first took advantage of all the data on clinical isolates available in the pubMLST database, and we clarified the spectrum of the epidemiology and extent of diversity of *V. parahaemolyticus* spreading throughout the world population. Although there were differences between the results obtained from the eBURST algorithm and the ME tree, the two analysis tools each have advantages. eBURST analysis can quickly find clonal complexes and their ancestral STs, while ME trees are better at revealing more evolutionary relationships among different STs. Applying the two models in simultaneous analyses can be conducive to arriving at more reliable conclusions. As increasing numbers of strains and STs are collected in the pubMLST database, more detailed information about the phylogenetic relationships of *V. parahaemolyticus* populations can be revealed in future studies.

In summary, the data reported in this study indicate that a high level of population genetic diversity exists in *V. parahaemolyticus*, and the *recA* gene appears to play a much greater role in the clonal diversification of this pathogen. The MLST scheme provides a universally available mechanism for timely recognition of both evolutionary trends and the emergence of *V. parahaemolyticus* clonal complexes. Broader application of this molecular epidemiology research method in the surveillance of *V. parahaemolyticus* infection outbreaks may be beneficial for preventing the large-scale spreading of virulent strains and for reducing the misuse of antibiotics for treatment as well as unnecessary costs.

## Supporting Information

Table S1 **Vibrio parahaemolyticus isolates used in this study (sorted by the timing of the identification of the strains).**
(DOC)

Table S2 **Genetic variation and evolutionary relationships of the STs in each clonal complex or doublet.**
(DOC)
