# Supplementary material for: Population Structure of Clinical Vibrio parahaemolyticus from 17 Coastal Countries, Determined through Multilocus Sequence Analysis
Source: PLoS One. 2014 Sep 16;9(9):e107371. doi: 10.1371/journal.pone.0107371 (PMC4165897; doi:10.1371/journal.pone.0107371)
Supplement: Table S1 — Vibrio parahaemolyticus isolates used in this study (sorted by the timing of the identification of the strains). (DOC) [file pone.0107371.s001.doc]

**Table S1**

***Vibrio parahaemolyticus* isolates used in this study (sorted by the timing of the identification of the strains).**

| ID | | Isolate | | Serovar | | STs | | Allele Numbers of the 7 Housekeeping Genes | | | | | | | | | | | | | | Times | | Country |
| --- | --- | --- | --- | --- | --- | --- | --- | --- | --- | --- | --- | --- | --- | --- | --- | --- | --- | --- | --- | --- | --- | --- | --- | --- |
|  |  |  |  |  |  |  |  | *dnaE* | | *gyrB* | | *recA* | | *dtdS* | | *pntA* | | *pyrC* | | *tnaA* | |  |  |  |
| 1 | | ATCC 17802 | | O1:K1 | | 1 | | 5 | | 52 | | 27 | | 13 | | 17 | | 25 | | 10 | | 1951 | | Japan |
| 2 | | S112_1416 | | Unknown* | | 1 | | 5 | | 52 | | 27 | | 13 | | 17 | | 25 | | 10 | | 1951 | | Japan |
| 3 | | S058_1395 | | Unknown | | 143 | | 95 | | 119 | | 93 | | 106 | | 74 | | 89 | | 70 | | 1970 | | Japan |
| 4 | | U5474 | | O3:K6 | | 14 | | 11 | | 4 | | 16 | | 35 | | 29 | | 15 | | 22 | | 1980 | | Bangladesh |
| 5 | | AQ3857 | | O1:K1 | | 83 | | 5 | | 52 | | 27 | | 13 | | 17 | | 25 | | 40 | | 1983 | | Japan |
| 6 | | AQ3860 | | O6:K46 | | 83 | | 5 | | 52 | | 27 | | 13 | | 17 | | 25 | | 40 | | 1983 | | Japan |
| 7 | | AQ3810 | | O3:K6 | | 87 | | 11 | | 4 | | 16 | | 35 | | 29 | | 52 | | 22 | | 1983 | | Japan |
| 8 | | AQ3855 | | O6:K18 | | 90 | | 29 | | 5 | | 22 | | 12 | | 20 | | 55 | | 25 | | 1983 | | Japan |
| 9 | | S010_1449 | | Unknown | | 91 | | 35 | | 4 | | 16 | | 50 | | 29 | | 5 | | 42 | | 1984 | | Japan |
| 10 | | S022_1442 | | Unknown | | 8 | | 28 | | 4 | | 82 | | 88 | | 63 | | 69 | | 1 | | 1984 | | Japan |
| 11 | | S031_1441 | | Unknown | | 189 | | 11 | | 48 | | 3 | | 48 | | 26 | | 48 | | 26 | | 1984 | | Japan |
| 12 | | S035_1445 | | Unknown | | 8 | | 28 | | 4 | | 82 | | 88 | | 63 | | 69 | | 1 | | 1984 | | Japan |
| 13 | | S105_1439 | | Unknown | | 325 | | 60 | | 104 | | 123 | | 47 | | 26 | | 69 | | 61 | | 1984 | | Japan |
| 14 | | S117_1452 | | Unknown | | 262 | | 105 | | 156 | | 123 | | 127 | | 19 | | 12 | | 47 | | 1984 | | Japan |
| 15 | | S123_1433 | | Unknown | | 217 | | 80 | | 88 | | 111 | | 109 | | 37 | | 95 | | 69 | | 1984 | | Japan |
| 16 | | S129_1446 | | Unknown | | 50 | | 29 | | 5 | | 22 | | 12 | | 20 | | 22 | | 25 | | 1984 | | Japan |
| 17 | | S031 | | O4:K13 | | 189 | | 11 | | 48 | | 3 | | 48 | | 26 | | 48 | | 26 | | 1984 | | Japan |
| 18 | | AQ4037 | | O3:K6 | | 96 | | 35 | | 4 | | 16 | | 50 | | 29 | | 66 | | 42 | | 1985 | | Japan |
| 19 | | S004_1240 | | Unknown | | 96 | | 35 | | 4 | | 16 | | 50 | | 29 | | 66 | | 42 | | 1985 | | Maldives |
| 20 | | S004 | | O3:K6 | | 96 | | 35 | | 4 | | 16 | | 50 | | 29 | | 66 | | 42 | | 1985 | | Maldives |
| 21 | | S008_1181 | | Unknown | | 91 | | 35 | | 4 | | 16 | | 50 | | 29 | | 5 | | 42 | | 1987 | | Thailand |
| 22 | | S009_1182 | | Unknown | | 91 | | 35 | | 4 | | 16 | | 50 | | 29 | | 5 | | 42 | | 1987 | | Thailand |
| 23 | | AQ4405 | | O3:K72 | | 83 | | 5 | | 52 | | 27 | | 13 | | 17 | | 25 | | 40 | | 1989 | | Japan |
| 24 | | AQ4433 | | O1:Kuk* | | 83 | | 5 | | 52 | | 27 | | 13 | | 17 | | 25 | | 40 | | 1989 | | Japan |
| 25 | | 48057 | | O4:K12 | | 36 | | 21 | | 15 | | 1 | | 23 | | 23 | | 21 | | 16 | | 1990 | | USA |
| 26 | | 48262 | | O1:K56 | | 44 | | 23 | | 15 | | 10 | | 7 | | 14 | | 24 | | 3 | | 1990 | | USA |
| 27 | | 48291 | | O12:K12 | | 36 | | 21 | | 15 | | 1 | | 23 | | 23 | | 21 | | 16 | | 1990 | | USA |
| 28 | | 275 | | O1:K1 | | 82 | | 5 | | 52 | | 27 | | 13 | | 18 | | 25 | | 40 | | 1990 | | Thailand |
| 29 | | VP172A | | O1:K1 | | 83 | | 5 | | 52 | | 27 | | 13 | | 17 | | 25 | | 40 | | 1990 | | Thailand |
| 30 | | VP172B | | O1:K2 | | 83 | | 5 | | 52 | | 27 | | 13 | | 17 | | 25 | | 40 | | 1990 | | Thailand |
| 31 | | VP176 | | O1:K1 | | 264 | | 5 | | 52 | | 27 | | 132 | | 17 | | 25 | | 40 | | 1990 | | Thailand |
| 32 | | VP180 | | O8:K22 | | 262 | | 105 | | 156 | | 123 | | 127 | | 19 | | 12 | | 47 | | 1990 | | Thailand |
| 33 | | VP182 | | O1:K69 | | 262 | | 105 | | 156 | | 123 | | 127 | | 19 | | 12 | | 47 | | 1990 | | Thailand |
| 34 | | VP184 | | O4:K11 | | 262 | | 105 | | 156 | | 123 | | 127 | | 19 | | 12 | | 47 | | 1990 | | Thailand |
| 35 | | VP190 | | O4:K10 | | 265 | | 11 | | 48 | | 107 | | 48 | | 26 | | 48 | | 26 | | 1990 | | Thailand |
| 36 | | VP200 | | O4:K8 | | 189 | | 11 | | 48 | | 3 | | 48 | | 26 | | 48 | | 26 | | 1990 | | Thailand |
| 37 | | S011_1477 | | Unknown | | 91 | | 35 | | 4 | | 16 | | 50 | | 29 | | 5 | | 42 | | 1990 | | Thailand |
| 38 | | S012_1475 | | Unknown | | 91 | | 35 | | 4 | | 16 | | 50 | | 29 | | 5 | | 42 | | 1990 | | Thailand |
| 39 | | S044_1481 | | Unknown | | 262 | | 105 | | 156 | | 123 | | 127 | | 19 | | 12 | | 47 | | 1990 | | Thailand |
| 40 | | S045_1482 | | Unknown | | 262 | | 105 | | 156 | | 123 | | 127 | | 19 | | 12 | | 47 | | 1990 | | Thailand |
| 41 | | S053_1487 | | Unknown | | 262 | | 105 | | 156 | | 123 | | 127 | | 19 | | 12 | | 47 | | 1990 | | Thailand |
| 42 | | S111_1479 | | Unknown | | 83 | | 5 | | 52 | | 27 | | 13 | | 17 | | 25 | | 40 | | 1990 | | Thailand |
| 43 | | S113_1483 | | Unknown | | 83 | | 5 | | 52 | | 27 | | 13 | | 17 | | 25 | | 40 | | 1990 | | India |
| 44 | | S120_1480 | | Unknown | | 327 | | 102 | | 67 | | 4 | | 79 | | 43 | | 63 | | 23 | | 1990 | | Thailand |
| 45 | | VP170 | | O3:Kuk | | 262 | | 105 | | 156 | | 123 | | 127 | | 19 | | 12 | | 47 | | 1991 | | Thailand |
| 46 | | VP174 | | O3:Kuk | | 263 | | 50 | | 55 | | 48 | | 52 | | 23 | | 53 | | 47 | | 1991 | | Thailand |
| 47 | | VP178 | | O1:K69 | | 262 | | 105 | | 156 | | 123 | | 127 | | 19 | | 12 | | 47 | | 1991 | | Thailand |
| 48 | | VP188 | | O1:K69 | | 262 | | 105 | | 156 | | 123 | | 127 | | 19 | | 12 | | 47 | | 1991 | | Thailand |
| 49 | | VP194 | | Ouk*:Kuk | | 83 | | 5 | | 52 | | 27 | | 13 | | 17 | | 25 | | 40 | | 1991 | | Thailand |
| 50 | | Vp901128 | | Unknown | | 135 | | 17 | | 16 | | 95 | | 7 | | 24 | | 16 | | 20 | | 1991 | | USA |
| 51 | | AQ4704 | | O1:Kuk | | 83 | | 5 | | 52 | | 27 | | 13 | | 17 | | 25 | | 40 | | 1992 | | Japan |
| 52 | | AQ4781 | | O1:Kuk | | 84 | | 49 | | 54 | | 46 | | 51 | | 18 | | 11 | | 8 | | 1992 | | Japan |
| 53 | | S014_133 | | Unknown | | 68 | | 41 | | 40 | | 36 | | 41 | | 36 | | 39 | | 32 | | 1992 | | China |
| 54 | | S015_134 | | Unknown | | 68 | | 41 | | 40 | | 36 | | 41 | | 36 | | 39 | | 32 | | 1992 | | China |
| 55 | | S016_166 | | Unknown | | 120 | | 60 | | 108 | | 86 | | 98 | | 18 | | 45 | | 51 | | 1992 | | China |
| 56 | | S060_196 | | Unknown | | 272 | | 60 | | 116 | | 91 | | 135 | | 50 | | 118 | | 2 | | 1992 | | China |
| 57 | | AQ4889 | | O4:K12 | | 86 | | 5 | | 53 | | 47 | | 28 | | 4 | | 51 | | 41 | | 1993 | | Japan |
| 58 | | AQ4901 | | O3:K6 | | 91 | | 35 | | 4 | | 16 | | 50 | | 29 | | 5 | | 42 | | 1993 | | Japan |
| 59 | | AQ4815 | | O1:K59 | | 84 | | 49 | | 54 | | 46 | | 51 | | 18 | | 11 | | 8 | | 1993 | | Japan |
| 60 | | S018_304 | | Unknown | | 120 | | 60 | | 108 | | 86 | | 98 | | 18 | | 45 | | 51 | | 1993 | | China |
| 61 | | S109_527 | | Unknown | | 326 | | 28 | | 28 | | 44 | | 46 | | 61 | | 49 | | 38 | | 1993 | | China |
| 62 | | WR1 | | Unknown | | 134 | | 92 | | 116 | | 25 | | 102 | | 28 | | 3 | | 20 | | 1993 | | USA |
| 63 | | AQ4969 | | O1:Kuk | | 83 | | 5 | | 52 | | 27 | | 13 | | 17 | | 25 | | 40 | | 1994 | | Japan |
| 64 | | AQ4966 | | O1:Kuk | | 83 | | 5 | | 52 | | 27 | | 13 | | 17 | | 25 | | 40 | | 1994 | | Japan |
| 65 | | S020_634 | | Unknown | | 326 | | 28 | | 28 | | 44 | | 46 | | 61 | | 49 | | 38 | | 1994 | | China |
| 66 | | S033_672 | | Unknown | | 8 | | 28 | | 4 | | 82 | | 88 | | 63 | | 69 | | 1 | | 1994 | | China |
| 67 | | S037_1389 | | Unknown | | 36 | | 21 | | 15 | | 1 | | 23 | | 23 | | 21 | | 16 | | 1994 | | USA |
| 68 | | S055_720 | | Unknown | | 69 | | 43 | | 41 | | 31 | | 42 | | 37 | | 40 | | 33 | | 1994 | | China |
| 69 | | S056_668 | | Unknown | | 69 | | 43 | | 41 | | 31 | | 42 | | 37 | | 40 | | 33 | | 1994 | | China |
| 70 | | S057_718 | | Unknown | | 69 | | 43 | | 41 | | 31 | | 42 | | 37 | | 40 | | 33 | | 1994 | | China |
| 71 | | 9546257 | | O4:K8 | | 32 | | 20 | | 25 | | 15 | | 6 | | 7 | | 11 | | 4 | | 1995 | | USA |
| 72 | | 324-95 | | O4:K8 | | 88 | | 11 | | 48 | | 43 | | 48 | | 26 | | 48 | | 26 | | 1995 | | Peru |
| 73 | | 326-95 | | O4:K8 | | 88 | | 11 | | 48 | | 43 | | 48 | | 26 | | 48 | | 26 | | 1995 | | Peru |
| 74 | | 288-95 | | O4:K8 | | 89 | | 28 | | 28 | | 44 | | 46 | | 40 | | 49 | | 38 | | 1995 | | Peru |
| 75 | | VP81 | | O3:K6 | | 3 | | 3 | | 4 | | 19 | | 4 | | 29 | | 4 | | 22 | | 1996 | | India |
| 76 | | VP86 | | O3:K6 | | 3 | | 3 | | 4 | | 19 | | 4 | | 29 | | 4 | | 22 | | 1996 | | India |
| 77 | | VP155 | | O3:K6 | | 3 | | 3 | | 4 | | 19 | | 4 | | 29 | | 4 | | 22 | | 1996 | | India |
| 78 | | VP96 | | O3:K6 | | 3 | | 3 | | 4 | | 19 | | 4 | | 29 | | 4 | | 22 | | 1996 | | India |
| 79 | | VPKX (RIMD 2210633) | | O3:K6 | | 3 | | 3 | | 4 | | 19 | | 4 | | 29 | | 4 | | 22 | | 1996 | | Japan |
| 80 | | ATCC BAA-239 | | O3:K6 | | 3 | | 3 | | 4 | | 19 | | 4 | | 29 | | 4 | | 22 | | 1996 | | India |
| 81 | | ATCC BAA-238 | | O3:K6 | | 3 | | 3 | | 4 | | 19 | | 4 | | 29 | | 4 | | 22 | | 1996 | | India |
| 82 | | 267-95 | | O4:K8 | | 88 | | 11 | | 48 | | 43 | | 48 | | 26 | | 48 | | 26 | | 1996 | | Peru |
| 83 | | VP164 | | O1:K58 | | 17 | | 13 | | 10 | | 19 | | 27 | | 28 | | 27 | | 21 | | 1996 | | Thailand |
| 84 | | VP166 | | O1:K1 | | 83 | | 5 | | 52 | | 27 | | 13 | | 17 | | 25 | | 40 | | 1996 | | Thailand |
| 85 | | VP168 | | O3:Kuk | | 66 | | 42 | | 25 | | 3 | | 40 | | 35 | | 38 | | 31 | | 1996 | | Thailand |
| 86 | | S013_1304 | | Unknown | | 91 | | 35 | | 4 | | 16 | | 50 | | 29 | | 5 | | 42 | | 1996 | | China |
| 87 | | S092_1291 | | Unknown | | 3 | | 3 | | 4 | | 19 | | 4 | | 29 | | 4 | | 22 | | 1996 | | China |
| 88 | | S094_1456 | | Unknown | | 3 | | 3 | | 4 | | 19 | | 4 | | 29 | | 4 | | 22 | | 1996 | | Thailand |
| 89 | | S095_1301 | | Unknown | | 91 | | 35 | | 4 | | 16 | | 50 | | 29 | | 5 | | 42 | | 1996 | | China |
| 90 | | Vp47 | | O3 : K6 | | 3 | | 3 | | 4 | | 19 | | 4 | | 29 | | 4 | | 22 | | 1996 | | India |
| 91 | | Peru-466 | | Unknown | | 3 | | 3 | | 4 | | 19 | | 4 | | 29 | | 4 | | 22 | | 1996 | | Peru |
| 92 | | 906-97 | | O3:K6 | | 3 | | 3 | | 4 | | 19 | | 4 | | 29 | | 4 | | 22 | | 1997 | | Peru |
| 93 | | 10296 | | O4:K12 | | 36 | | 21 | | 15 | | 1 | | 23 | | 23 | | 21 | | 16 | | 1997 | | USA |
| 94 | | 10290 | | O4:K12 | | 37 | | 21 | | 15 | | 1 | | 23 | | 23 | | 20 | | 16 | | 1997 | | USA |
| 95 | | 10292 | | O6:K18 | | 50 | | 29 | | 5 | | 22 | | 12 | | 20 | | 22 | | 25 | | 1997 | | USA |
| 96 | | VP208 | | O3:K6 | | 3 | | 3 | | 4 | | 19 | | 4 | | 29 | | 4 | | 22 | | 1997 | | India |
| 97 | | 219 | | O6:Kuk | | 81 | | 45 | | 45 | | 40 | | 7 | | 14 | | 46 | | 36 | | 1997 | | Norway (probably) |
| 98 | | 763-97 | | O3:K6 | | 3 | | 3 | | 4 | | 19 | | 4 | | 29 | | 4 | | 22 | | 1997 | | Peru |
| 99 | | S066_1152 | | Unknown | | 3 | | 3 | | 4 | | 19 | | 4 | | 29 | | 4 | | 22 | | 1997 | | China |
| 100 | | S067_1153 | | Unknown | | 3 | | 3 | | 4 | | 19 | | 4 | | 29 | | 4 | | 22 | | 1997 | | China |
| 101 | | S068_1155 | | Unknown | | 3 | | 3 | | 4 | | 19 | | 4 | | 29 | | 4 | | 22 | | 1997 | | China |
| 102 | | S074_1139 | | Unknown | | 3 | | 3 | | 4 | | 19 | | 4 | | 29 | | 4 | | 22 | | 1997 | | China |
| 103 | | S104_1405 | | Unknown | | 50 | | 29 | | 5 | | 22 | | 12 | | 20 | | 22 | | 25 | | 1997 | | USA |
| 104 | | 901128 | | Unknown | | 135 | | 17 | | 16 | | 95 | | 7 | | 24 | | 16 | | 20 | | 1997 | | USA |
| 105 | | 97-10290 | | Unknown | | 43 | | 23 | | 29 | | 10 | | 7 | | 14 | | 24 | | 2 | | 1997 | | USA |
| 106 | | EN9701072 | | Unknown | | 43 | | 23 | | 29 | | 10 | | 7 | | 14 | | 24 | | 2 | | 1997 | | USA |
| 107 | | EN9701121 | | Unknown | | 50 | | 29 | | 5 | | 22 | | 12 | | 20 | | 22 | | 25 | | 1997 | | USA |
| 108 | | EN9701173 | | Unknown | | 36 | | 21 | | 15 | | 1 | | 23 | | 23 | | 21 | | 16 | | 1997 | | USA |
| 109 | | NY477 | | Unknown | | 88 | | 11 | | 48 | | 43 | | 48 | | 26 | | 48 | | 26 | | 1997 | | USA |
| 110 | | SPRC10290 | | Unknown | | 36 | | 21 | | 15 | | 1 | | 23 | | 23 | | 21 | | 16 | | 1997 | | USA |
| 111 | | VP-NY4 | | Unknown | | 3 | | 3 | | 4 | | 19 | | 4 | | 29 | | 4 | | 22 | | 1997 | | India |
| 112 | | 428/00 | | O4:K11 | | 17 | | 13 | | 10 | | 19 | | 27 | | 28 | | 27 | | 21 | | 1998 | | Spain |
| 113 | | 10329 | | O4:K12 | | 36 | | 21 | | 15 | | 1 | | 23 | | 23 | | 21 | | 16 | | 1998 | | USA |
| 114 | | AN-8373 | | O3:K6 | | 3 | | 3 | | 4 | | 19 | | 4 | | 29 | | 4 | | 22 | | 1998 | | Bangladesh |
| 115 | | JYKVP6 | | O3:K6 | | 3 | | 3 | | 4 | | 19 | | 4 | | 29 | | 4 | | 22 | | 1998 | | Japan |
| 116 | | VP2 | | O3:K6 | | 27 | | 17 | | 4 | | 19 | | 4 | | 29 | | 4 | | 22 | | 1998 | | Korea |
| 117 | | NY-3483 | | O4:K12 | | 36 | | 21 | | 15 | | 1 | | 23 | | 23 | | 21 | | 16 | | 1998 | | USA |
| 118 | | TX2103 | | O3:K6 | | 3 | | 3 | | 4 | | 19 | | 4 | | 29 | | 4 | | 22 | | 1998 | | USA |
| 119 | | BAC-98-3372 | | O3:K6 | | 3 | | 3 | | 4 | | 19 | | 4 | | 29 | | 4 | | 22 | | 1998 | | USA |
| 120 | | BAC-98-3374 | | O3:K6 | | 42 | | 22 | | 4 | | 19 | | 4 | | 29 | | 4 | | 22 | | 1998 | | USA |
| 121 | | BAC-98-4092 | | O3:K6 | | 3 | | 3 | | 4 | | 19 | | 4 | | 29 | | 4 | | 22 | | 1998 | | USA |
| 122 | | AN-5034 | | O4:K68 | | 3 | | 3 | | 4 | | 19 | | 4 | | 29 | | 4 | | 22 | | 1998 | | Bangladesh |
| 123 | | AN-16000 | | O1:Kuk | | 3 | | 3 | | 4 | | 19 | | 4 | | 29 | | 4 | | 22 | | 1998 | | Bangladesh |
| 124 | | KXV-641 | | O1:K25 | | 3 | | 3 | | 4 | | 19 | | 4 | | 29 | | 4 | | 22 | | 1998 | | Japan |
| 125 | | AN-2189 | | O4:K68 | | 3 | | 3 | | 4 | | 19 | | 4 | | 29 | | 4 | | 22 | | 1998 | | Bangladesh |
| 126 | | ATCC BAA-241 | | O4:K68 | | 3 | | 3 | | 4 | | 19 | | 4 | | 29 | | 4 | | 22 | | 1998 | | India |
| 127 | | ATCC BAA-242 | | O1:Kuk | | 3 | | 3 | | 4 | | 19 | | 4 | | 29 | | 4 | | 22 | | 1998 | | India |
| 128 | | AN-7410 | | O3:K6 | | 3 | | 3 | | 4 | | 19 | | 4 | | 29 | | 4 | | 22 | | 1998 | | Bangladesh |
| 129 | | AN-11790 | | O4:K68 | | 3 | | 3 | | 4 | | 19 | | 4 | | 29 | | 4 | | 22 | | 1998 | | Bangladesh |
| 130 | | 3435-98 | | O3:K6 | | 3 | | 3 | | 4 | | 19 | | 4 | | 29 | | 4 | | 22 | | 1998 | | Peru |
| 131 | | 784-98 | | O3:K6 | | 3 | | 3 | | 4 | | 19 | | 4 | | 29 | | 4 | | 22 | | 1998 | | Peru |
| 132 | | ATC210 | | O3:K6 | | 3 | | 3 | | 4 | | 19 | | 4 | | 29 | | 4 | | 22 | | 1998 | | Chile |
| 133 | | ATC220 | | O3:K6 | | 3 | | 3 | | 4 | | 19 | | 4 | | 29 | | 4 | | 22 | | 1998 | | Chile |
| 134 | | FIHES98V1-32-4 | | O3:K6 | | 3 | | 3 | | 4 | | 19 | | 4 | | 29 | | 4 | | 22 | | 1998 | | Japan |
| 135 | | S032_1261 | | Unknown | | 8 | | 28 | | 4 | | 82 | | 88 | | 63 | | 69 | | 1 | | 1998 | | Phillipine |
| 136 | | S061_1262 | | Unknown | | 3 | | 3 | | 4 | | 19 | | 4 | | 29 | | 4 | | 22 | | 1998 | | Singapore |
| 137 | | S062_1263 | | Unknown | | 3 | | 3 | | 4 | | 19 | | 4 | | 29 | | 4 | | 22 | | 1998 | | Singapore |
| 138 | | S063_1346 | | Unknown | | 3 | | 3 | | 4 | | 19 | | 4 | | 29 | | 4 | | 22 | | 1998 | | China |
| 139 | | S064_1347 | | Unknown | | 3 | | 3 | | 4 | | 19 | | 4 | | 29 | | 4 | | 22 | | 1998 | | China |
| 140 | | S065_1267 | | Unknown | | 3 | | 3 | | 4 | | 19 | | 4 | | 29 | | 4 | | 22 | | 1998 | | China |
| 141 | | S071_1247 | | Unknown | | 3 | | 3 | | 4 | | 19 | | 4 | | 29 | | 4 | | 22 | | 1998 | | Bangladesh |
| 142 | | S072_1248 | | Unknown | | 3 | | 3 | | 4 | | 19 | | 4 | | 29 | | 4 | | 22 | | 1998 | | Bangladesh |
| 143 | | S083_1249 | | Unknown | | 3 | | 3 | | 4 | | 19 | | 4 | | 29 | | 4 | | 22 | | 1998 | | Japan |
| 144 | | S087_1264 | | Unknown | | 3 | | 3 | | 4 | | 19 | | 4 | | 29 | | 4 | | 22 | | 1998 | | Singapore |
| 145 | | S088_1264 | | Unknown | | 3 | | 3 | | 4 | | 19 | | 4 | | 29 | | 4 | | 22 | | 1998 | | Singapore |
| 146 | | S089_1265 | | Unknown | | 3 | | 3 | | 4 | | 19 | | 4 | | 29 | | 4 | | 22 | | 1998 | | Singapore |
| 147 | | S093_1252 | | Unknown | | 3 | | 3 | | 4 | | 19 | | 4 | | 29 | | 4 | | 22 | | 1998 | | Japan |
| 148 | | S114_1484 | | Unknown | | 83 | | 5 | | 52 | | 27 | | 13 | | 17 | | 25 | | 40 | | 1998 | | India |
| 149 | | AN7410 | | O3:K6 | | 3 | | 3 | | 4 | | 19 | | 4 | | 29 | | 4 | | 22 | | 1998 | | Bangladesh |
| 150 | | ATCC BAA-241 | | O4:K68 | | 3 | | 3 | | 4 | | 19 | | 4 | | 29 | | 4 | | 22 | | 1998 | | India |
| 151 | | AN11790 | | O4:K68 | | 3 | | 3 | | 4 | | 19 | | 4 | | 29 | | 4 | | 22 | | 1998 | | Bangladesh |
| 152 | | ATCC BAA-242 | | O1:KUT | | 3 | | 3 | | 4 | | 19 | | 4 | | 29 | | 4 | | 22 | | 1998 | | India |
| 153 | | BE98-2029 | | Unknown | | 3 | | 3 | | 4 | | 19 | | 4 | | 29 | | 4 | | 22 | | 1998 | | USA |
| 154 | | VP232 | | Unknown | | 3 | | 3 | | 4 | | 19 | | 4 | | 29 | | 4 | | 22 | | 1998 | | India |
| 155 | | VP250 | | Unknown | | 3 | | 3 | | 4 | | 19 | | 4 | | 29 | | 4 | | 22 | | 1998 | | India |
| 156 | | 30824 | | O4:K11 | | 17 | | 13 | | 10 | | 19 | | 27 | | 28 | | 27 | | 21 | | 1999 | | Spain |
| 157 | | 357-99 | | O3:Kuk | | 19 | | 15 | | 11 | | 30 | | 10 | | 1 | | 3 | | 9 | | 1999 | | Peru |
| 158 | | AO-24491 | | O1:K25 | | 3 | | 3 | | 4 | | 19 | | 4 | | 29 | | 4 | | 22 | | 1999 | | Bangladesh |
| 159 | | VPHY191 | | O1:K25 | | 3 | | 3 | | 4 | | 19 | | 4 | | 29 | | 4 | | 22 | | 1999 | | Thailand |
| 160 | | VPHY145 | | O4:K68 | | 3 | | 3 | | 4 | | 19 | | 4 | | 29 | | 4 | | 22 | | 1999 | | Thailand |
| 161 | | 220 | | O3:K6 | | 71 | | 3 | | 4 | | 4 | | 4 | | 29 | | 4 | | 22 | | 1999 | | Ecuador |
| 162 | | 222 | | O3:K6 | | 3 | | 3 | | 4 | | 19 | | 4 | | 29 | | 4 | | 22 | | 1999 | | Ecuador |
| 163 | | 221 | | O3:Kuk | | 80 | | 48 | | 5 | | 41 | | 43 | | 31 | | 45 | | 22 | | 1999 | | Norway (probably) |
| 164 | | 275-99 | | O3:K58 | | 3 | | 3 | | 4 | | 19 | | 4 | | 29 | | 4 | | 22 | | 1999 | | Peru |
| 165 | | 276-99 | | O3:K6 | | 3 | | 3 | | 4 | | 19 | | 4 | | 29 | | 4 | | 22 | | 1999 | | Peru |
| 166 | | 278-99 | | O3:K6 | | 3 | | 3 | | 4 | | 19 | | 4 | | 29 | | 4 | | 22 | | 1999 | | Peru |
| 167 | | 279-99 | | O3:K6 | | 3 | | 3 | | 4 | | 19 | | 4 | | 29 | | 4 | | 22 | | 1999 | | Peru |
| 168 | | 698-99 | | O3:K6 | | 3 | | 3 | | 4 | | 19 | | 4 | | 29 | | 4 | | 22 | | 1999 | | Peru |
| 169 | | S002_1293 | | Unknown | | 216 | | 98 | | 135 | | 112 | | 107 | | 77 | | 97 | | 26 | | 1999 | | China |
| 170 | | S021_1373 | | Unknown | | 326 | | 28 | | 28 | | 44 | | 46 | | 61 | | 49 | | 38 | | 1999 | | India |
| 171 | | S030_1379 | | Unknown | | 189 | | 11 | | 48 | | 3 | | 48 | | 26 | | 48 | | 26 | | 1999 | | India |
| 172 | | S034_1381 | | Unknown | | 8 | | 28 | | 4 | | 82 | | 88 | | 63 | | 69 | | 1 | | 1999 | | India |
| 173 | | S075_1227 | | Unknown | | 3 | | 3 | | 4 | | 19 | | 4 | | 29 | | 4 | | 22 | | 1999 | | China |
| 174 | | S076_1228 | | Unknown | | 3 | | 3 | | 4 | | 19 | | 4 | | 29 | | 4 | | 22 | | 1999 | | China |
| 175 | | S077_1229 | | Unknown | | 3 | | 3 | | 4 | | 19 | | 4 | | 29 | | 4 | | 22 | | 1999 | | China |
| 176 | | S078_1230 | | Unknown | | 3 | | 3 | | 4 | | 19 | | 4 | | 29 | | 4 | | 22 | | 1999 | | China |
| 177 | | S086_1259 | | Unknown | | 3 | | 3 | | 4 | | 19 | | 4 | | 29 | | 4 | | 22 | | 1999 | | Thailand |
| 178 | | S090_1362 | | Unknown | | 3 | | 3 | | 4 | | 19 | | 4 | | 29 | | 4 | | 22 | | 1999 | | China |
| 179 | | S091_1377 | | Unknown | | 3 | | 3 | | 4 | | 19 | | 4 | | 29 | | 4 | | 22 | | 1999 | | India |
| 180 | | S096_1283 | | Unknown | | 217 | | 80 | | 88 | | 111 | | 109 | | 37 | | 95 | | 69 | | 1999 | | Korea |
| 181 | | S110_1380 | | Unknown | | 83 | | 5 | | 52 | | 27 | | 13 | | 17 | | 25 | | 40 | | 1999 | | India |
| 182 | | S119_1364 | | Unknown | | 224 | | 28 | | 83 | | 82 | | 117 | | 18 | | 69 | | 79 | | 1999 | | China |
| 183 | | S122_1376 | | Unknown | | 217 | | 80 | | 88 | | 111 | | 109 | | 37 | | 95 | | 69 | | 1999 | | India |
| 184 | | EN9901310 | | Unknown | | 36 | | 21 | | 15 | | 1 | | 23 | | 23 | | 21 | | 16 | | 1999 | | USA |
| 185 | | S030 | | O4:K8 | | 189 | | 11 | | 48 | | 3 | | 48 | | 26 | | 48 | | 26 | | 1999 | | India |
| 186 | | S002 | | O2:K3 | | 216 | | 98 | | 135 | | 112 | | 107 | | 77 | | 97 | | 26 | | 1999 | | China |
| 187 | | AP-11243 | | O1:Kuk | | 51 | | 29 | | 4 | | 19 | | 4 | | 29 | | 4 | | 22 | | 2000 | | Bangladesh |
| 188 | | 330-00 | | O3:K6 | | 3 | | 3 | | 4 | | 19 | | 4 | | 29 | | 4 | | 22 | | 2000 | | Peru |
| 189 | | 405-00 | | O3:K6 | | 3 | | 3 | | 4 | | 19 | | 4 | | 29 | | 4 | | 22 | | 2000 | | Peru |
| 190 | | 429-00 | | O3:K6 | | 3 | | 3 | | 4 | | 19 | | 4 | | 29 | | 4 | | 22 | | 2000 | | Peru |
| 191 | | 430-00 | | O3:K6 | | 3 | | 3 | | 4 | | 19 | | 4 | | 29 | | 4 | | 22 | | 2000 | | Peru |
| 192 | | 461-00 | | O3:K6 | | 3 | | 3 | | 4 | | 19 | | 4 | | 29 | | 4 | | 22 | | 2000 | | Peru |
| 193 | | 462-00 | | O3:K6 | | 3 | | 3 | | 4 | | 19 | | 4 | | 29 | | 4 | | 22 | | 2000 | | Peru |
| 194 | | 511-00 | | O3:K6 | | 3 | | 3 | | 4 | | 19 | | 4 | | 29 | | 4 | | 22 | | 2000 | | Peru |
| 195 | | 512-00 | | O3:K6 | | 3 | | 3 | | 4 | | 19 | | 4 | | 29 | | 4 | | 22 | | 2000 | | Peru |
| 196 | | 776-00 | | O6:Kuk | | 93 | | 51 | | 50 | | 31 | | 29 | | 41 | | 46 | | 39 | | 2000 | | Peru |
| 197 | | EN2910 | | Unknown | | 36 | | 21 | | 15 | | 1 | | 23 | | 23 | | 21 | | 16 | | 2000 | | USA |
| 198 | | HC-01-22 | | O4:K63 | | 43 | | 23 | | 29 | | 10 | | 7 | | 14 | | 24 | | 2 | | 2001 | | USA |
| 199 | | 224 | | O3:Kuk | | 34 | | 20 | | 25 | | 15 | | 13 | | 7 | | 11 | | 5 | | 2001 | | Norway (probably) |
| 200 | | 169-02 | | O3:K6 | | 3 | | 3 | | 4 | | 19 | | 4 | | 29 | | 4 | | 22 | | 2001 | | Peru |
| 201 | | 056-01 | | O3:K6 | | 3 | | 3 | | 4 | | 19 | | 4 | | 29 | | 4 | | 22 | | 2001 | | Peru |
| 202 | | 498-01 | | O3:K6 | | 3 | | 3 | | 4 | | 19 | | 4 | | 29 | | 4 | | 22 | | 2001 | | Peru |
| 203 | | 565-01 | | O3:K6 | | 3 | | 3 | | 4 | | 19 | | 4 | | 29 | | 4 | | 22 | | 2001 | | Peru |
| 204 | | 568-01 | | O3:K6 | | 3 | | 3 | | 4 | | 19 | | 4 | | 29 | | 4 | | 22 | | 2001 | | Peru |
| 205 | | 463-01 | | O1:K33 | | 94 | | 52 | | 49 | | 45 | | 49 | | 4 | | 50 | | 23 | | 2001 | | Peru |
| 206 | | Peru-288 | | Unknown | | 3 | | 3 | | 4 | | 19 | | 4 | | 29 | | 4 | | 22 | | 2001 | | Peru |
| 207 | | 225 | | O3:Kuk | | 72 | | 3 | | 4 | | 4 | | 4 | | 29 | | 4 | | 22 | | 2002 | | Thailand |
| 208 | | 226 | | O3:Kuk | | 3 | | 3 | | 4 | | 19 | | 4 | | 29 | | 4 | | 22 | | 2002 | | Thailand |
| 209 | | 227 | | O5:K68 | | 3 | | 3 | | 4 | | 19 | | 4 | | 29 | | 4 | | 22 | | 2002 | | Norway (probably) |
| 210 | | 228 | | O11:Kuk | | 73 | | 3 | | 44 | | 42 | | 44 | | 38 | | 44 | | 24 | | 2002 | | Norway (probably) |
| 211 | | 004-02 | | O3:K6 | | 3 | | 3 | | 4 | | 19 | | 4 | | 29 | | 4 | | 22 | | 2002 | | Peru |
| 212 | | 020-02 | | O3:K6 | | 3 | | 3 | | 4 | | 19 | | 4 | | 29 | | 4 | | 22 | | 2002 | | Peru |
| 213 | | 240-02 | | O3:K6 | | 3 | | 3 | | 4 | | 19 | | 4 | | 29 | | 4 | | 22 | | 2002 | | Peru |
| 214 | | 551-02 | | O3:K6 | | 3 | | 3 | | 4 | | 19 | | 4 | | 29 | | 4 | | 22 | | 2002 | | Peru |
| 215 | | 552-02 | | O3:K6 | | 3 | | 3 | | 4 | | 19 | | 4 | | 29 | | 4 | | 22 | | 2002 | | Peru |
| 216 | | 038-03 | | O3:K6 | | 3 | | 3 | | 4 | | 19 | | 4 | | 29 | | 4 | | 22 | | 2003 | | Peru |
| 217 | | 039-03 | | Unknown | | 3 | | 3 | | 4 | | 19 | | 4 | | 29 | | 4 | | 22 | | 2003 | | Peru |
| 218 | | 131-03 | | O3:K6 | | 3 | | 3 | | 4 | | 19 | | 4 | | 29 | | 4 | | 22 | | 2003 | | Peru |
| 219 | | 302-03 | | O3:K6 | | 3 | | 3 | | 4 | | 19 | | 4 | | 29 | | 4 | | 22 | | 2003 | | Peru |
| 220 | | S130_Shen | | Unknown | | 69 | | 43 | | 41 | | 31 | | 42 | | 37 | | 40 | | 33 | | 2003 | | China |
| 221 | | S131_Qin | | Unknown | | 3 | | 3 | | 4 | | 19 | | 4 | | 29 | | 4 | | 22 | | 2003 | | China |
| 222 | | S135_ICDC-VP77 | | Unknown | | 3 | | 3 | | 4 | | 19 | | 4 | | 29 | | 4 | | 22 | | 2003 | | China |
| 223 | | S130 | | Unknown | | 527 | | 43 | | 41 | | 107 | | 42 | | 37 | | 40 | | 33 | | 2003 | | China |
| 224 | | Jan-08 | | O3:K6 | | 3 | | 3 | | 4 | | 19 | | 4 | | 29 | | 4 | | 22 | | 2004 | | Spain |
| 225 | | IB3882 | | O3:K58 | | 66 | | 42 | | 25 | | 3 | | 40 | | 35 | | 38 | | 31 | | 2004 | | Mozambique |
| 226 | | IB3883 | | O3:Kuk | | 67 | | 44 | | 42 | | 35 | | 29 | | 26 | | 41 | | 13 | | 2004 | | Mozambique |
| 227 | | IB3927 | | O8:K41 | | 69 | | 43 | | 41 | | 31 | | 42 | | 37 | | 40 | | 33 | | 2004 | | Mozambique |
| 228 | | IB3884 | | O3:K58 | | 66 | | 42 | | 25 | | 3 | | 40 | | 35 | | 38 | | 31 | | 2004 | | Mozambique |
| 229 | | IB3885 | | O3:K58 | | 66 | | 42 | | 25 | | 3 | | 40 | | 35 | | 38 | | 31 | | 2004 | | Mozambique |
| 230 | | IB3886 | | O3:K58 | | 66 | | 42 | | 25 | | 3 | | 40 | | 35 | | 38 | | 31 | | 2004 | | Mozambique |
| 231 | | IB3887 | | O4:K68 | | 3 | | 3 | | 4 | | 19 | | 4 | | 29 | | 4 | | 22 | | 2004 | | Mozambique |
| 232 | | IB3888 | | O3:K58 | | 66 | | 42 | | 25 | | 3 | | 40 | | 35 | | 38 | | 31 | | 2004 | | Mozambique |
| 233 | | IB3889 | | O3:K6 | | 3 | | 3 | | 4 | | 19 | | 4 | | 29 | | 4 | | 22 | | 2004 | | Mozambique |
| 234 | | IB3890 | | O4:K68 | | 3 | | 3 | | 4 | | 19 | | 4 | | 29 | | 4 | | 22 | | 2004 | | Mozambique |
| 235 | | IB3891 | | O3:K58 | | 66 | | 42 | | 25 | | 3 | | 40 | | 35 | | 38 | | 31 | | 2004 | | Mozambique |
| 236 | | IB3892 | | O3:K6 | | 3 | | 3 | | 4 | | 19 | | 4 | | 29 | | 4 | | 22 | | 2004 | | Mozambique |
| 237 | | IB3897 | | O4:K13 | | 68 | | 41 | | 40 | | 36 | | 41 | | 36 | | 39 | | 32 | | 2004 | | Mozambique |
| 238 | | IB3898 | | O3:K6 | | 3 | | 3 | | 4 | | 19 | | 4 | | 29 | | 4 | | 22 | | 2004 | | Mozambique |
| 239 | | IB3904 | | O3:K6 | | 3 | | 3 | | 4 | | 19 | | 4 | | 29 | | 4 | | 22 | | 2004 | | Mozambique |
| 240 | | IB3910 | | O3:K6 | | 3 | | 3 | | 4 | | 19 | | 4 | | 29 | | 4 | | 22 | | 2004 | | Mozambique |
| 241 | | IB3916 | | O3:K6 | | 3 | | 3 | | 4 | | 19 | | 4 | | 29 | | 4 | | 22 | | 2004 | | Mozambique |
| 242 | | IB3922 | | O3:K6 | | 3 | | 3 | | 4 | | 19 | | 4 | | 29 | | 4 | | 22 | | 2004 | | Mozambique |
| 243 | | PMC39.5 | | O3:K6 | | 3 | | 3 | | 4 | | 19 | | 4 | | 29 | | 4 | | 22 | | 2004 | | Chile |
| 244 | | PMC42.5 | | O3:K6 | | 3 | | 3 | | 4 | | 19 | | 4 | | 29 | | 4 | | 22 | | 2004 | | Chile |
| 245 | | PMC48.4 | | O3:K6 | | 3 | | 3 | | 4 | | 19 | | 4 | | 29 | | 4 | | 22 | | 2004 | | Chile |
| 246 | | S136_ICDC-VP87 | | Unknown | | 3 | | 3 | | 4 | | 19 | | 4 | | 29 | | 4 | | 22 | | 2004 | | China |
| 247 | | B-265 | | Unknown | | 3 | | 3 | | 4 | | 19 | | 4 | | 29 | | 4 | | 22 | | 2004 | | Mozambique |
| 248 | | IB3925 | | O4:K13 | | 68 | | 41 | | 40 | | 36 | | 41 | | 36 | | 39 | | 32 | | 2005 | | Mozambique |
| 249 | | IB3928 | | O4:K13 | | 68 | | 41 | | 40 | | 36 | | 41 | | 36 | | 39 | | 32 | | 2005 | | Mozambique |
| 250 | | IB3929 | | O4:K13 | | 68 | | 41 | | 40 | | 36 | | 41 | | 36 | | 39 | | 32 | | 2005 | | Mozambique |
| 251 | | IB3931 | | O3:K6 | | 3 | | 3 | | 4 | | 19 | | 4 | | 29 | | 4 | | 22 | | 2005 | | Mozambique |
| 252 | | IB3932 | | O3:K58 | | 66 | | 42 | | 25 | | 3 | | 40 | | 35 | | 38 | | 31 | | 2005 | | Mozambique |
| 253 | | IB3933 | | O4:K13 | | 68 | | 41 | | 40 | | 36 | | 41 | | 36 | | 39 | | 32 | | 2005 | | Mozambique |
| 254 | | IB3934 | | O4:K68 | | 3 | | 3 | | 4 | | 19 | | 4 | | 29 | | 4 | | 22 | | 2005 | | Mozambique |
| 255 | | IB3935 | | O3:K58 | | 66 | | 42 | | 25 | | 3 | | 40 | | 35 | | 38 | | 31 | | 2005 | | Mozambique |
| 256 | | IB3936 | | O4:K13 | | 68 | | 41 | | 40 | | 36 | | 41 | | 36 | | 39 | | 32 | | 2005 | | Mozambique |
| 257 | | IB3937 | | O3:K6 | | 3 | | 3 | | 4 | | 19 | | 4 | | 29 | | 4 | | 22 | | 2005 | | Mozambique |
| 258 | | 205-05 | | O1:Kuk | | 3 | | 3 | | 4 | | 19 | | 4 | | 29 | | 4 | | 22 | | 2005 | | Peru |
| 259 | | 553-02 | | O3:K6 | | 3 | | 3 | | 4 | | 19 | | 4 | | 29 | | 4 | | 22 | | 2005 | | Peru |
| 260 | | 155-05 | | O1:Kuk | | 65 | | 39 | | 9 | | 27 | | 39 | | 3 | | 37 | | 30 | | 2005 | | Peru |
| 261 | | 156-05 | | O1:Kuk | | 65 | | 39 | | 9 | | 27 | | 39 | | 3 | | 37 | | 30 | | 2005 | | Peru |
| 262 | | S132_ICDC-VP32 | | Unknown | | 328 | | 14 | | 30 | | 139 | | 78 | | 4 | | 37 | | 13 | | 2005 | | China |
| 263 | | S133_ICDC-VP53 | | Unknown | | 3 | | 3 | | 4 | | 19 | | 4 | | 29 | | 4 | | 22 | | 2005 | | China |
| 264 | | S134_ICDC-VP75 | | Unknown | | 69 | | 43 | | 41 | | 31 | | 42 | | 37 | | 40 | | 33 | | 2005 | | China |
| 265 | | S137_ICDC-VP88 | | Unknown | | 3 | | 3 | | 4 | | 19 | | 4 | | 29 | | 4 | | 22 | | 2005 | | China |
| 266 | | K5030 | | Unknown | | 3 | | 3 | | 4 | | 19 | | 4 | | 29 | | 4 | | 22 | | 2005 | | India |
| 267 | | S134 | | Unknown | | 527 | | 43 | | 41 | | 107 | | 42 | | 37 | | 40 | | 33 | | 2005 | | China |
| 268 | | V249 | | O4:Kuk | | 128 | | 38 | | 25 | | 19 | | 96 | | 45 | | 81 | | 65 | | 2006 | | China |
| 269 | | V251 | | O3:K6 | | 3 | | 3 | | 4 | | 19 | | 4 | | 29 | | 4 | | 22 | | 2006 | | China |
| 270 | | V262 | | O3:K33 | | 127 | | 47 | | 112 | | 38 | | 97 | | 68 | | 69 | | 64 | | 2006 | | China |
| 271 | | V264 | | O3:K6 | | 3 | | 3 | | 4 | | 19 | | 4 | | 29 | | 4 | | 22 | | 2006 | | China |
| 272 | | V267 | | O1:Kuk | | 3 | | 3 | | 4 | | 19 | | 4 | | 29 | | 4 | | 22 | | 2006 | | China |
| 273 | | V263 | | O3:K68 | | 3 | | 3 | | 4 | | 19 | | 4 | | 29 | | 4 | | 22 | | 2006 | | China |
| 274 | | V258 | | O3:K68 | | 3 | | 3 | | 4 | | 19 | | 4 | | 29 | | 4 | | 22 | | 2006 | | China |
| 275 | | V261 | | O3:K6 | | 120 | | 60 | | 108 | | 86 | | 98 | | 18 | | 45 | | 51 | | 2006 | | China |
| 276 | | V254 | | O3:Kuk | | 189 | | 11 | | 48 | | 3 | | 48 | | 26 | | 48 | | 26 | | 2006 | | China |
| 277 | | V405 | | O5:K17 | | 194 | | 97 | | 127 | | 101 | | 29 | | 78 | | 99 | | 47 | | 2006 | | China |
| 278 | | P15 | | O4:KUT | | 301 | | 140 | | 167 | | 136 | | 151 | | 50 | | 135 | | 17 | | 2006 | | China |
| 279 | | S003_1185 | | Unknown | | 91 | | 35 | | 4 | | 16 | | 50 | | 29 | | 5 | | 42 | | 2006 | | China |
| 280 | | S005_1186 | | Unknown | | 91 | | 35 | | 4 | | 16 | | 50 | | 29 | | 5 | | 42 | | 2006 | | Thailand |
| 281 | | S026_1465 | | Unknown | | 17 | | 13 | | 10 | | 19 | | 27 | | 28 | | 27 | | 21 | | 2006 | | Thailand |
| 282 | | S027_1410 | | Unknown | | 17 | | 13 | | 10 | | 19 | | 27 | | 28 | | 27 | | 21 | | 2006 | | USA |
| 283 | | S028_1466 | | Unknown | | 328 | | 14 | | 30 | | 139 | | 78 | | 4 | | 37 | | 13 | | 2006 | | Thailand |
| 284 | | S036_1461 | | Unknown | | 8 | | 28 | | 4 | | 82 | | 88 | | 63 | | 69 | | 1 | | 2006 | | Thailand |
| 285 | | S039_1036 | | Unknown | | 321 | | 12 | | 101 | | 84 | | 91 | | 58 | | 78 | | 33 | | 2006 | | China |
| 286 | | S054_1467 | | Unknown | | 69 | | 43 | | 41 | | 31 | | 42 | | 37 | | 40 | | 33 | | 2006 | | Thailand |
| 287 | | S069_1463 | | Unknown | | 3 | | 3 | | 4 | | 19 | | 4 | | 29 | | 4 | | 22 | | 2006 | | Thailand |
| 288 | | S070_1468 | | Unknown | | 3 | | 3 | | 4 | | 19 | | 4 | | 29 | | 4 | | 22 | | 2006 | | Thailand |
| 289 | | S079_1221 | | Unknown | | 3 | | 3 | | 4 | | 19 | | 4 | | 29 | | 4 | | 22 | | 2006 | | Indonesia |
| 290 | | S081_1203 | | Unknown | | 3 | | 3 | | 4 | | 19 | | 4 | | 29 | | 4 | | 22 | | 2006 | | Korea |
| 291 | | S082_1458 | | Unknown | | 3 | | 3 | | 4 | | 19 | | 4 | | 29 | | 4 | | 22 | | 2006 | | Thailand |
| 292 | | S126_1188 | | Unknown | | 3 | | 3 | | 4 | | 19 | | 4 | | 29 | | 4 | | 22 | | 2006 | | China |
| 293 | | 12298 | | Unknown | | 36 | | 21 | | 15 | | 1 | | 23 | | 23 | | 21 | | 16 | | 2006 | | USA |
| 294 | | 12310 | | Unknown | | 36 | | 21 | | 15 | | 1 | | 23 | | 23 | | 21 | | 16 | | 2006 | | USA |
| 295 | | 12315 | | Unknown | | 36 | | 21 | | 15 | | 1 | | 23 | | 23 | | 21 | | 16 | | 2006 | | USA |
| 296 | | 12317 | | Unknown | | 36 | | 21 | | 15 | | 1 | | 23 | | 23 | | 21 | | 16 | | 2006 | | USA |
| 297 | | 12355 | | Unknown | | 36 | | 21 | | 15 | | 1 | | 23 | | 23 | | 21 | | 16 | | 2006 | | USA |
| 298 | | 12383 | | Unknown | | 36 | | 21 | | 15 | | 1 | | 23 | | 23 | | 21 | | 16 | | 2006 | | USA |
| 299 | | 12447 | | Unknown | | 36 | | 21 | | 15 | | 1 | | 23 | | 23 | | 21 | | 16 | | 2006 | | USA |
| 300 | | 12601 | | Unknown | | 43 | | 23 | | 29 | | 10 | | 7 | | 14 | | 24 | | 2 | | 2006 | | USA |
| 301 | | VP06157 | | O8:K22 | | 529 | | 112 | | 266 | | 190 | | 12 | | 39 | | 73 | | 86 | | 2006 | | China |
| 302 | FDA_R31/CDC_K4557 | | O1:KUT | | 23 | | 17 | | 16 | | 13 | | 36 | | 15 | | 31 | | 26 | | 2006 | | USA | |
| 303 | 949 | | Unknown | | 3 | | 3 | | 4 | | 19 | | 4 | | 29 | | 4 | | 22 | | 2006 | | USA | |
| 304 | NIHCB0603 | | Unknown | | 3 | | 3 | | 4 | | 19 | | 4 | | 29 | | 4 | | 22 | | 2006 | | Bangladesh | |
| 305 | NIHCB0757 | | Unknown | | 65 | | 39 | | 9 | | 27 | | 39 | | 3 | | 37 | | 30 | | 2006 | | Bangladesh | |
| 306 | 1 | | O1:K25 | | 3 | | 3 | | 4 | | 19 | | 4 | | 29 | | 4 | | 22 | | 2006 | | China | |
| 307 | W1 | | O1:K25 | | 3 | | 3 | | 4 | | 19 | | 4 | | 29 | | 4 | | 22 | | 2006 | | China | |
| 308 | 34 | | O1:K25 | | 3 | | 3 | | 4 | | 19 | | 4 | | 29 | | 4 | | 22 | | 2006 | | China | |
| 309 | 3 | | O3:K6 | | 3 | | 3 | | 4 | | 19 | | 4 | | 29 | | 4 | | 22 | | 2006 | | China | |
| 310 | 50 | | Unknown | | 34 | | 20 | | 25 | | 15 | | 13 | | 7 | | 11 | | 5 | | 2006 | | USA | |
| 311 | PMC 38.7 | | Unknown | | 63 | | 7 | | 38 | | 34 | | 27 | | 28 | | 8 | | 2 | | 2007 | | Chile | |
| 312 | PMC 53.7 | | Unknown | | 28 | | 17 | | 16 | | 13 | | 7 | | 24 | | 16 | | 20 | | 2007 | | Chile | |
| 313 | PMC 60.7 | | Unknown | | 64 | | 40 | | 39 | | 3 | | 18 | | 4 | | 36 | | 29 | | 2007 | | Chile | |
| 314 | 301-07 | | O1:Kuk | | 3 | | 3 | | 4 | | 19 | | 4 | | 29 | | 4 | | 22 | | 2007 | | Peru | |
| 315 | 304-07 | | O3:K30 | | 3 | | 3 | | 4 | | 19 | | 4 | | 29 | | 4 | | 22 | | 2007 | | Peru | |
| 316 | 369-07 | | O3:Kuk | | 3 | | 3 | | 4 | | 19 | | 4 | | 29 | | 4 | | 22 | | 2007 | | Peru | |
| 317 | 371-07 | | O1:Kuk | | 3 | | 3 | | 4 | | 19 | | 4 | | 29 | | 4 | | 22 | | 2007 | | Peru | |
| 318 | 438-07 | | O3:Kuk | | 3 | | 3 | | 4 | | 19 | | 4 | | 29 | | 4 | | 22 | | 2007 | | Peru | |
| 319 | PMC29.7 | | O3:K6 | | 3 | | 3 | | 4 | | 19 | | 4 | | 29 | | 4 | | 22 | | 2007 | | Chile | |
| 320 | PMC55.7 | | O3:K6 | | 3 | | 3 | | 4 | | 19 | | 4 | | 29 | | 4 | | 22 | | 2007 | | Chile | |
| 321 | PMC73.7 | | O3:K6 | | 3 | | 3 | | 4 | | 19 | | 4 | | 29 | | 4 | | 22 | | 2007 | | Chile | |
| 322 | 437-07 | | O3:Kuk | | 64 | | 40 | | 39 | | 3 | | 18 | | 4 | | 36 | | 29 | | 2007 | | Peru | |
| 323 | V248 | | O3:K29 | | 188 | | 60 | | 108 | | 86 | | 99 | | 18 | | 45 | | 51 | | 2007 | | China | |
| 324 | V275 | | O1:K36 | | 3 | | 3 | | 4 | | 19 | | 4 | | 29 | | 4 | | 22 | | 2007 | | China | |
| 325 | V276 | | O3:K6 | | 3 | | 3 | | 4 | | 19 | | 4 | | 29 | | 4 | | 22 | | 2007 | | China | |
| 326 | V283 | | O3:K25 | | 3 | | 3 | | 4 | | 19 | | 4 | | 29 | | 4 | | 22 | | 2007 | | China | |
| 327 | V253 | | O10:Kuk | | 129 | | 49 | | 107 | | 90 | | 19 | | 73 | | 37 | | 20 | | 2007 | | China | |
| 328 | V327 | | O1:K26 | | 192 | | 3 | | 126 | | 19 | | 4 | | 29 | | 4 | | 22 | | 2007 | | China | |
| 329 | V336 | | O3:K6 | | 3 | | 3 | | 4 | | 19 | | 4 | | 29 | | 4 | | 22 | | 2007 | | China | |
| 330 | P202 | | O4: K8 | | 283 | | 27 | | 84 | | 127 | | 139 | | 54 | | 124 | | 37 | | 2007 | | China | |
| 331 | P43 | | O4:KUT | | 302 | | 27 | | 106 | | 127 | | 152 | | 54 | | 124 | | 101 | | 2007 | | China | |
| 332 | S138_ICDC-VP133 | | Unknown | | 3 | | 3 | | 4 | | 19 | | 4 | | 29 | | 4 | | 22 | | 2007 | | China | |
| 333 | PMC50.7 | | O3:K6 | | 3 | | 3 | | 4 | | 19 | | 4 | | 29 | | 4 | | 22 | | 2007 | | Chile | |
| 334 | PMC51.7 | | O3:K6 | | 3 | | 3 | | 4 | | 19 | | 4 | | 29 | | 4 | | 22 | | 2007 | | Chile | |
| 335 | PMC41.7 | | O3:K6 | | 3 | | 3 | | 4 | | 19 | | 4 | | 29 | | 4 | | 22 | | 2007 | | Chile | |
| 336 | PMC72.7 | | O3:K6 | | 3 | | 3 | | 4 | | 19 | | 4 | | 29 | | 4 | | 22 | | 2007 | | Chile | |
| 337 | PMC1.7 | | O3:K6 | | 3 | | 3 | | 4 | | 19 | | 4 | | 29 | | 4 | | 22 | | 2007 | | Chile | |
| 338 | PMC15.7 | | O3:K6 | | 3 | | 3 | | 4 | | 19 | | 4 | | 29 | | 4 | | 22 | | 2007 | | Chile | |
| 339 | PMC16.7 | | O3:K6 | | 3 | | 3 | | 4 | | 19 | | 4 | | 29 | | 4 | | 22 | | 2007 | | Chile | |
| 340 | PMC38.7 | | O10:K20 | | 63 | | 7 | | 38 | | 34 | | 27 | | 28 | | 8 | | 2 | | 2007 | | Chile | |
| 341 | PMC47.7 | | O10:K20 | | 63 | | 7 | | 38 | | 34 | | 27 | | 28 | | 8 | | 2 | | 2007 | | Chile | |
| 342 | PMC57.7 | | O10:K20 | | 63 | | 7 | | 38 | | 34 | | 27 | | 28 | | 8 | | 2 | | 2007 | | Chile | |
| 343 | PMC68.7 | | O10:K20 | | 63 | | 7 | | 38 | | 34 | | 27 | | 28 | | 8 | | 2 | | 2007 | | Chile | |
| 344 | PMC60.7 | | O1:KUT | | 64 | | 40 | | 39 | | 3 | | 18 | | 4 | | 36 | | 29 | | 2007 | | Chile | |
| 345 | PMC25.7 | | O1:KUT | | 64 | | 40 | | 39 | | 3 | | 18 | | 4 | | 36 | | 29 | | 2007 | | Chile | |
| 346 | PMC26.7 | | O1:KUT | | 64 | | 40 | | 39 | | 3 | | 18 | | 4 | | 36 | | 29 | | 2007 | | Chile | |
| 347 | PMC27.7 | | O1:KUT | | 64 | | 40 | | 39 | | 3 | | 18 | | 4 | | 36 | | 29 | | 2007 | | Chile | |
| 348 | PMC53.7 | | O3:K59 | | 28 | | 17 | | 16 | | 13 | | 7 | | 24 | | 16 | | 20 | | 2007 | | Chile | |
| 349 | PMC75.7 | | O1:KUT | | 65 | | 39 | | 9 | | 27 | | 39 | | 3 | | 37 | | 30 | | 2007 | | Chile | |
| 350 | 3256 | | Unknown | | 36 | | 21 | | 15 | | 1 | | 23 | | 23 | | 21 | | 16 | | 2007 | | USA | |
| 351 | 3259 | | Unknown | | 65 | | 39 | | 9 | | 27 | | 39 | | 3 | | 37 | | 30 | | 2007 | | USA | |
| 352 | 3270 | | Unknown | | 36 | | 21 | | 15 | | 1 | | 23 | | 23 | | 21 | | 16 | | 2007 | | USA | |
| 353 | 3271 | | Unknown | | 36 | | 21 | | 15 | | 1 | | 23 | | 23 | | 21 | | 16 | | 2007 | | USA | |
| 354 | 3324 | | Unknown | | 36 | | 21 | | 15 | | 1 | | 23 | | 23 | | 21 | | 16 | | 2007 | | USA | |
| 355 | 3326 | | Unknown | | 36 | | 21 | | 15 | | 1 | | 23 | | 23 | | 21 | | 16 | | 2007 | | USA | |
| 356 | 3328 | | Unknown | | 65 | | 39 | | 9 | | 27 | | 39 | | 3 | | 37 | | 30 | | 2007 | | USA | |
| 357 | 3355 | | Unknown | | 65 | | 39 | | 9 | | 27 | | 39 | | 3 | | 37 | | 30 | | 2007 | | USA | |
| 358 | 3631 | | Unknown | | 417 | | 3 | | 111 | | 167 | | 188 | | 116 | | 167 | | 33 | | 2007 | | USA | |
| 359 | 3644 | | Unknown | | 43 | | 23 | | 29 | | 10 | | 7 | | 14 | | 24 | | 2 | | 2007 | | USA | |
| 360 | 3646 | | Unknown | | 417 | | 3 | | 111 | | 167 | | 188 | | 116 | | 167 | | 33 | | 2007 | | USA | |
| 361 | VP07250 | | O1:K25 | | 481 | | 4 | | 13 | | 11 | | 91 | | 18 | | 9 | | 23 | | 2007 | | China | |
| 362 | 07VP054 | | O1:K38 | | 777 | | 51 | | 104 | | 178 | | 146 | | 28 | | 45 | | 12 | | 2007 | | China | |
| 363 | 3259 | | Unknown | | 479 | | 186 | | 252 | | 181 | | 29 | | 4 | | 18 | | 132 | | 2007 | | USA | |
| 364 | VP2007-095 | | Unknown | | 631 | | 222 | | 128 | | 21 | | 69 | | 46 | | 236 | | 12 | | 2007 | | USA | |
| 365 | HY20 | | O11:K36 | | 3 | | 3 | | 4 | | 19 | | 4 | | 29 | | 4 | | 22 | | 2007 | | China | |
| 366 | HY19 | | O11:K36 | | 3 | | 3 | | 4 | | 19 | | 4 | | 29 | | 4 | | 22 | | 2007 | | China | |
| 367 | W50 | | O11:K36 | | 3 | | 3 | | 4 | | 19 | | 4 | | 29 | | 4 | | 22 | | 2007 | | China | |
| 368 | VIP4 | | Unknown | | 3 | | 3 | | 4 | | 19 | | 4 | | 29 | | 4 | | 22 | | 2007 | | China | |
| 369 | 3256 | | Unknown | | 36 | | 21 | | 15 | | 1 | | 23 | | 23 | | 21 | | 16 | | 2007 | | USA | |
| 370 | V485 | | O1:K56 | | 123 | | 35 | | 111 | | 85 | | 100 | | 26 | | 84 | | 1 | | 2008 | | China | |
| 371 | V495 | | O1:K56 | | 8 | | 28 | | 4 | | 82 | | 88 | | 63 | | 69 | | 1 | | 2008 | | China | |
| 372 | V502 | | O1:K56 | | 8 | | 28 | | 4 | | 82 | | 88 | | 63 | | 69 | | 1 | | 2008 | | China | |
| 373 | V381 | | O3:Kuk | | 193 | | 44 | | 130 | | 87 | | 110 | | 29 | | 37 | | 63 | | 2008 | | China | |
| 374 | V501 | | O1:Kuk | | 200 | | 103 | | 3 | | 89 | | 3 | | 72 | | 82 | | 2 | | 2008 | | China | |
| 375 | V350 | | O3:K6 | | 3 | | 3 | | 4 | | 19 | | 4 | | 29 | | 4 | | 22 | | 2008 | | China | |
| 376 | V377 | | O1:K56 | | 8 | | 28 | | 4 | | 82 | | 88 | | 63 | | 69 | | 1 | | 2008 | | China | |
| 377 | V378 | | O3:K6 | | 216 | | 98 | | 135 | | 112 | | 107 | | 77 | | 97 | | 26 | | 2008 | | China | |
| 378 | V472 | | O1:K38 | | 217 | | 80 | | 88 | | 111 | | 109 | | 37 | | 95 | | 69 | | 2008 | | China | |
| 379 | V625 | | O1:K36 | | 223 | | 108 | | 136 | | 109 | | 35 | | 11 | | 82 | | 24 | | 2008 | | China | |
| 380 | ATCC 33847 | | O4:K11 | | 224 | | 28 | | 83 | | 82 | | 117 | | 18 | | 69 | | 79 | | 2008 | | USA | |
| 381 | V357 | | O2:K29 | | 120 | | 60 | | 108 | | 86 | | 98 | | 18 | | 45 | | 51 | | 2008 | | China | |
| 382 | V252 | | O4:K68 | | 3 | | 3 | | 4 | | 19 | | 4 | | 29 | | 4 | | 22 | | 2008 | | China | |
| 383 | VP11153 | | O3:K6 | | 487 | | 3 | | 48 | | 19 | | 4 | | 29 | | 4 | | 22 | | 2008 | | China | |
| 384 | VP11155 | | O3:K6 | | 489 | | 3 | | 4 | | 19 | | 4 | | 29 | | 197 | | 22 | | 2008 | | China | |
| 385 | VP07015 | | O1:K25 | | 491 | | 28 | | 106 | | 82 | | 204 | | 18 | | 7 | | 26 | | 2008 | | China | |
| 386 | F08-36 | | O5:K17 | | 781 | | 31 | | 132 | | 73 | | 13 | | 4 | | 145 | | 1 | | 2008 | | China | |
| 387 | F08-92 | | O1:K56 | | 783 | | 28 | | 4 | | 82 | | 88 | | 63 | | 140 | | 1 | | 2008 | | China | |
| 388 | VP08353 | | O1:K56 | | 812 | | 11 | | 48 | | 82 | | 48 | | 26 | | 48 | | 26 | | 2008 | | China | |
| 389 | VP08354 | | O1:K56 | | 812 | | 11 | | 48 | | 82 | | 48 | | 26 | | 48 | | 26 | | 2008 | | China | |
| 390 | V-11 | | O1:K25 | | 305 | | 3 | | 4 | | 19 | | 147 | | 93 | | 4 | | 22 | | 2008 | | China | |
| 391 | V-12 | | O1:K25 | | 305 | | 3 | | 4 | | 19 | | 147 | | 93 | | 4 | | 22 | | 2008 | | China | |
| 392 | 3' | | O11:K36 | | 3 | | 3 | | 4 | | 19 | | 4 | | 29 | | 4 | | 22 | | 2008 | | China | |
| 393 | BA2 | | O3:K6 | | 3 | | 3 | | 4 | | 19 | | 4 | | 29 | | 4 | | 22 | | 2008 | | China | |
| 394 | GM6 | | O4:K68 | | 3 | | 3 | | 4 | | 19 | | 4 | | 29 | | 4 | | 22 | | 2008 | | China | |
| 395 | GM8 | | O4:K68 | | 3 | | 3 | | 4 | | 19 | | 4 | | 29 | | 4 | | 22 | | 2008 | | China | |
| 396 | VIP-0439 | | Unknown | | 3 | | 3 | | 4 | | 19 | | 4 | | 29 | | 4 | | 22 | | 2008 | | China | |
| 397 | V456 | | O4:K37 | | 62 | | 19 | | 4 | | 88 | | 2 | | 34 | | 18 | | 23 | | 2009 | | China | |
| 398 | V478 | | O1:Kuk | | 199 | | 22 | | 28 | | 17 | | 13 | | 8 | | 19 | | 14 | | 2009 | | China | |
| 399 | V504 | | O3:K6 | | 3 | | 3 | | 4 | | 19 | | 4 | | 29 | | 4 | | 22 | | 2009 | | China | |
| 400 | V473 | | O10:KUT | | 218 | | 100 | | 123 | | 110 | | 13 | | 11 | | 96 | | 57 | | 2009 | | China | |
| 401 | V529 | | O1:Kuk | | 226 | | 28 | | 90 | | 113 | | 82 | | 81 | | 104 | | 26 | | 2009 | | China | |
| 402 | V533 | | O1:Kuk | | 199 | | 22 | | 28 | | 17 | | 13 | | 8 | | 19 | | 14 | | 2009 | | China | |
| 403 | V506 | | Unknown | | 227 | | 3 | | 4 | | 19 | | 4 | | 29 | | 22 | | 22 | | 2009 | | China | |
| 404 | V562 | | O1:K28 | | 303 | | 125 | | 160 | | 89 | | 13 | | 18 | | 82 | | 76 | | 2009 | | China | |
| 405 | VP09332 | | O1:K41 | | 483 | | 39 | | 113 | | 27 | | 39 | | 3 | | 37 | | 61 | | 2009 | | China | |
| 406 | VP09019 | | O3:K1 | | 493 | | 5 | | 36 | | 27 | | 13 | | 18 | | 192 | | 77 | | 2009 | | China | |
| 407 | VP09094 | | O8:K21 | | 494 | | 186 | | 258 | | 181 | | 29 | | 4 | | 18 | | 132 | | 2009 | | China | |
| 408 | VP09122 | | O8:K21 | | 494 | | 186 | | 258 | | 181 | | 29 | | 4 | | 18 | | 132 | | 2009 | | China | |
| 409 | VP09433 | | O6:K18 | | 495 | | 192 | | 36 | | 166 | | 212 | | 26 | | 193 | | 38 | | 2009 | | China | |
| 410 | VP09075 | | O5:K17 | | 530 | | 11 | | 106 | | 192 | | 220 | | 4 | | 73 | | 17 | | 2009 | | China | |
| 411 | VP09157 | | O5:K17 | | 532 | | 93 | | 77 | | 144 | | 19 | | 23 | | 201 | | 23 | | 2009 | | China | |
| 412 | VP09059 | | O4:K12 | | 813 | | 33 | | 261 | | 93 | | 151 | | 176 | | 52 | | 194 | | 2009 | | China | |
| 413 | IDH02189 | | Unknown | | 3 | | 3 | | 4 | | 19 | | 4 | | 29 | | 4 | | 22 | | 2009 | | India | |
| 414 | IDH02640 | | Unknown | | 3 | | 3 | | 4 | | 19 | | 4 | | 29 | | 4 | | 22 | | 2009 | | India | |
| 415 | 735 | | Unknown | | 330 | | 146 | | 180 | | 19 | | 88 | | 73 | | 11 | | 51 | | 2010 | | China | |
| 416 | 736 | | Unknown | | 331 | | 147 | | 181 | | 127 | | 69 | | 26 | | 18 | | 23 | | 2010 | | China | |
| 417 | Vp0022 | | Unknown | | 332 | | 14 | | 30 | | 141 | | 78 | | 4 | | 37 | | 13 | | 2010 | | China | |
| 418 | Vp0030 | | Unknown | | 333 | | 60 | | 108 | | 31 | | 98 | | 18 | | 45 | | 51 | | 2010 | | China | |
| 419 | VP026 | | Unknown | | 338 | | 149 | | 184 | | 31 | | 76 | | 98 | | 11 | | 84 | | 2010 | | China | |
| 420 | VP032 | | Unknown | | 337 | | 47 | | 139 | | 53 | | 19 | | 50 | | 143 | | 26 | | 2010 | | China | |
| 421 | VP040 | | Unknown | | 338 | | 149 | | 184 | | 31 | | 76 | | 98 | | 11 | | 84 | | 2010 | | China | |
| 422 | VP023 | | Unknown | | 341 | | 28 | | 4 | | 127 | | 88 | | 63 | | 69 | | 1 | | 2010 | | China | |
| 423 | VP041 | | Unknown | | 340 | | 98 | | 4 | | 136 | | 107 | | 77 | | 46 | | 33 | | 2010 | | China | |
| 424 | VP009 | | Unknown | | 343 | | 4 | | 181 | | 19 | | 4 | | 29 | | 4 | | 22 | | 2010 | | China | |
| 425 | VP011 | | Unknown | | 344 | | 147 | | 181 | | 127 | | 69 | | 26 | | 18 | | 22 | | 2010 | | China | |
| 426 | VP013 | | Unknown | | 345 | | 11 | | 48 | | 19 | | 48 | | 26 | | 48 | | 26 | | 2010 | | China | |
| 427 | Hangzhou2010-25 | | O3:K17 | | 675 | | 31 | | 316 | | 25 | | 274 | | 71 | | 73 | | 62 | | 2010 | | China | |
| 428 | 82 | | Unknown | | 431 | | 3 | | 225 | | 19 | | 4 | | 29 | | 4 | | 22 | | 2010 | | China | |
| 429 | 87 | | Unknown | | 432 | | 33 | | 211 | | 31 | | 167 | | 118 | | 172 | | 17 | | 2010 | | China | |
| 430 | 85 | | Unknown | | 479 | | 186 | | 252 | | 181 | | 29 | | 4 | | 18 | | 132 | | 2010 | | China | |
| 431 | VP10133 | | O1:K56 | | 482 | | 28 | | 4 | | 82 | | 4 | | 63 | | 69 | | 1 | | 2010 | | China | |
| 432 | VP10140 | | O1:K36 | | 492 | | 3 | | 4 | | 189 | | 4 | | 29 | | 4 | | 22 | | 2010 | | China | |
| 433 | VP10368 | | O11:K36 | | 496 | | 3 | | 4 | | 19 | | 4 | | 29 | | 4 | | 142 | | 2010 | | China | |
| 434 | VP10339 | | O3:K41 | | 527 | | 43 | | 41 | | 107 | | 42 | | 37 | | 40 | | 33 | | 2010 | | China | |
| 435 | HZ2010-94 | | O3:Kuk | | 654 | | 35 | | 110 | | 29 | | 78 | | 10 | | 86 | | 51 | | 2010 | | China | |
| 436 | Hangzhou2010-10 | | O4:K18 | | 670 | | 106 | | 315 | | 31 | | 29 | | 158 | | 11 | | 174 | | 2010 | | China | |
| 437 | F10-68 | | O4:Kuk | | 776 | | 49 | | 314 | | 38 | | 219 | | 26 | | 203 | | 26 | | 2010 | | China | |
| 438 | F10-74 | | O5:KUT | | 784 | | 35 | | 43 | | 38 | | 21 | | 31 | | 37 | | 37 | | 2010 | | China | |
| 439 | F10-105 | | O4:K68 | | 787 | | 3 | | 4 | | 19 | | 4 | | 29 | | 48 | | 22 | | 2010 | | China | |
| 440 | F10-106 | | O4:K68 | | 787 | | 3 | | 4 | | 19 | | 4 | | 29 | | 48 | | 22 | | 2010 | | China | |
| 441 | F10-69 | | O2:K3 | | 788 | | 98 | | 4 | | 30 | | 32 | | 77 | | 48 | | 82 | | 2010 | | China | |
| 442 | F10-43 | | O4:K9 | | 789 | | 14 | | 30 | | 141 | | 78 | | 4 | | 35 | | 13 | | 2010 | | China | |
| 443 | F10-38 | | O2:K28 | | 791 | | 5 | | 41 | | 217 | | 198 | | 26 | | 48 | | 48 | | 2010 | | China | |
| 444 | VP10053 | | O4:K12 | | 814 | | 27 | | 226 | | 73 | | 27 | | 26 | | 18 | | 37 | | 2010 | | China | |
| 445 | VP10162 | | O1:K56 | | 815 | | 71 | | 345 | | 246 | | 167 | | 177 | | 69 | | 47 | | 2010 | | China | |
| 446 | VP10190 | | O3:K29 | | 816 | | 3 | | 111 | | 167 | | 188 | | 116 | | 5 | | 33 | | 2010 | | China | |
| 447 | VP10191 | | O3:K6 | | 3 | | 3 | | 4 | | 19 | | 4 | | 29 | | 4 | | 22 | | 2010 | | China | |
| 448 | SH11VP69 | | O10 | | 443 | | 133 | | 234 | | 70 | | 75 | | 28 | | 11 | | 99 | | 2011 | | China | |
| 449 | SH11VP70 | | O4:K34 | | 444 | | 47 | | 58 | | 19 | | 19 | | 50 | | 37 | | 26 | | 2011 | | China | |
| 450 | SH11VP75 | | O10 | | 448 | | 133 | | 67 | | 4 | | 79 | | 43 | | 63 | | 62 | | 2011 | | China | |
| 451 | SH11VP4 | | O4 | | 472 | | 7 | | 236 | | 99 | | 26 | | 6 | | 18 | | 26 | | 2011 | | China | |
| 452 | SH11VP28 | | O10 | | 475 | | 172 | | 227 | | 171 | | 181 | | 119 | | 5 | | 57 | | 2011 | | China | |
| 453 | SH11VP32 | | O2 | | 476 | | 96 | | 228 | | 31 | | 3 | | 120 | | 131 | | 33 | | 2011 | | China | |
| 454 | SH11VP38 | | O10 | | 477 | | 172 | | 227 | | 170 | | 181 | | 119 | | 5 | | 57 | | 2011 | | China | |
| 455 | SH11VP40 | | O2:K3 | | 433 | | 98 | | 4 | | 112 | | 107 | | 77 | | 97 | | 23 | | 2011 | | China | |
| 456 | SH11VP47 | | O2 | | 434 | | 98 | | 229 | | 112 | | 107 | | 18 | | 97 | | 23 | | 2011 | | China | |
| 457 | SH11VP48 | | O3:K6 | | 435 | | 3 | | 4 | | 31 | | 4 | | 29 | | 4 | | 22 | | 2011 | | China | |
| 458 | 162 | | Unknown | | 478 | | 185 | | 226 | | 73 | | 19 | | 26 | | 18 | | 37 | | 2011 | | China | |
| 459 | VP11093 | | O4:K8 | | 438 | | 11 | | 108 | | 19 | | 48 | | 26 | | 48 | | 26 | | 2011 | | China | |
| 460 | VP11095 | | O3:K29 | | 480 | | 60 | | 4 | | 86 | | 98 | | 18 | | 45 | | 51 | | 2011 | | China | |
| 461 | VP11134 | | O3:K29 | | 480 | | 60 | | 4 | | 86 | | 98 | | 18 | | 45 | | 51 | | 2011 | | China | |
| 462 | VP11036 | | O1:K41 | | 484 | | 43 | | 41 | | 19 | | 42 | | 37 | | 40 | | 33 | | 2011 | | China | |
| 463 | VP11228 | | O3:K6 | | 497 | | 5 | | 137 | | 123 | | 50 | | 23 | | 196 | | 79 | | 2011 | | China | |
| 464 | VP11133 | | O3:K6 | | 526 | | 3 | | 108 | | 19 | | 4 | | 29 | | 4 | | 22 | | 2011 | | China | |
| 465 | 2011 37-11 | | O8:K41 | | 806 | | 244 | | 41 | | 107 | | 42 | | 37 | | 40 | | 33 | | 2011 | | China | |
| 466 | Hangzhou2012-191 | | O1:Kuk | | 671 | | 35 | | 110 | | 227 | | 78 | | 10 | | 86 | | 51 | | 2012 | | China | |
| 467 | Hangzhou2010-599 | | O3:K6 | | 672 | | 1 | | 147 | | 19 | | 4 | | 29 | | 4 | | 22 | | 2012 | | China | |
| 468 | VP16MD | | Unknown | | 3 | | 3 | | 4 | | 19 | | 4 | | 29 | | 4 | | 22 | | 2012 | | USA | |
| 469 | VP17MD | | Unknown | | 3 | | 3 | | 4 | | 19 | | 4 | | 29 | | 4 | | 22 | | 2012 | | USA | |
| 470 | VP18MD | | Unknown | | 3 | | 3 | | 4 | | 19 | | 4 | | 29 | | 4 | | 22 | | 2012 | | USA | |
| 471 | 2012 29 | | O2:K3 | | 216 | | 98 | | 135 | | 112 | | 107 | | 77 | | 97 | | 26 | | 2012 | | China | |
| 472 | 2012 8-11 | | O1 | | 807 | | 176 | | 197 | | 245 | | 298 | | 6 | | 39 | | 66 | | 2012 | | China | |
| 473 | VP12010 | | O1:K56 | | 817 | | 48 | | 5 | | 41 | | 299 | | 140 | | 7 | | 108 | | 2012 | | China | |
| 474 | VP12121 | | O4:K12 | | 813 | | 33 | | 261 | | 93 | | 151 | | 176 | | 52 | | 194 | | 2012 | | China | |
| 475 | VP12136 | | O8:K21 | | 818 | | 245 | | 346 | | 239 | | 300 | | 19 | | 269 | | 99 | | 2012 | | China | |
| 476 | VP19 | | Unknown | | 863 | | 2 | | 198 | | 72 | | 94 | | 26 | | 7 | | 94 | | 2012 | | China | |
| 477 | VP25 | | Unknown | | 886 | | 51 | | 4 | | 19 | | 4 | | 29 | | 4 | | 22 | | 2012 | | China | |
| 478 | VP33 | | Unknown | | 887 | | 137 | | 198 | | 144 | | 82 | | 18 | | 152 | | 86 | | 2012 | | China | |
| 479 | VP40Lux | | Unknown | | 888 | | 28 | | 39 | | 230 | | 19 | | 61 | | 195 | | 1 | | 2012 | | China | |
| 480 | VP43 | | Unknown | | 889 | | 92 | | 106 | | 74 | | 102 | | 28 | | 3 | | 20 | | 2012 | | China | |
| 481 | VP44Lux | | Unknown | | 890 | | 92 | | 106 | | 25 | | 102 | | 28 | | 3 | | 20 | | 2012 | | China | |
| 482 | VP45 | | Unknown | | 891 | | 208 | | 300 | | 213 | | 76 | | 147 | | 46 | | 24 | | 2012 | | China | |
| 483 | VP46Lux | | Unknown | | 891 | | 208 | | 300 | | 213 | | 76 | | 147 | | 46 | | 24 | | 2012 | | China | |
| 484 | VP47 | | Unknown | | 891 | | 208 | | 300 | | 213 | | 76 | | 147 | | 46 | | 24 | | 2012 | | China | |
| 485 | VP50Lux | | Unknown | | 892 | | 8 | | 168 | | 98 | | 13 | | 56 | | 45 | | 57 | | 2012 | | China | |
| 486 | VP53 | | Unknown | | 893 | | 10 | | 58 | | 62 | | 95 | | 50 | | 85 | | 2 | | 2012 | | China | |
| 487 | VP54Lux | | Unknown | | 894 | | 35 | | 269 | | 101 | | 167 | | 80 | | 79 | | 57 | | 2012 | | China | |
| 488 | VP58Lux | | Unknown | | 895 | | 80 | | 43 | | 49 | | 74 | | 26 | | 7 | | 34 | | 2012 | | China | |
| 489 | FX-185 | | O3:K6 | | 3 | | 3 | | 4 | | 19 | | 4 | | 29 | | 4 | | 22 | | 2012 | | China | |
| 490 | V-213 | | O4:K68 | | 3 | | 3 | | 4 | | 19 | | 4 | | 29 | | 4 | | 22 | | 2012 | | China | |

*OuK, Kuk and Unknown: not detected or not collected.
