# Supplementary material for: Population Structure of Clinical Vibrio parahaemolyticus from 17 Coastal Countries, Determined through Multilocus Sequence Analysis
Source: PLoS One. 2014 Sep 16;9(9):e107371. doi: 10.1371/journal.pone.0107371 (PMC4165897; doi:10.1371/journal.pone.0107371)
Supplement: Table S2 — Genetic variation and evolutionary relationships of the STs in each clonal complex or doublet. (DOC) [file pone.0107371.s002.doc]

**Table S2**

Genetic variation and evolutionary relationships of the STs in each clonal complex or doublet

| Clonal complexes / Doublets | STs | Allele Numbers of the 7 Housekeeping Genes | | | | | | | Phylogenetic analysis by ME tree |
| --- | --- | --- | --- | --- | --- | --- | --- | --- | --- |
|  |  | *dnaE* | *gyrB* | *recA* | *dtdS* | *pntA* | *pyrC* | *tnaA* |  |
| **CC3** |  |  |  |  |  |  |  |  |  |
|  | ST3 | 3 | 4 | 19 | 4 | 29 | 4 | 22 | ST435, ST71 and ST72 reflect a distant evolutionary relationship from CC3 |
|  | ST492 | 3 | 4 | 189 | 4 | 29 | 4 | 22 |  |
|  | ST192 | 3 | 126 | 19 | 4 | 29 | 4 | 22 |  |
|  | ST526 | 3 | 108 | 19 | 4 | 29 | 4 | 22 |  |
|  | ST487 | 3 | 48 | 19 | 4 | 29 | 4 | 22 |  |
|  | ST431 | 3 | 225 | 19 | 4 | 29 | 4 | 22 |  |
|  | ST51 | 29 | 4 | 19 | 4 | 29 | 4 | 22 |  |
|  | ST42 | 22 | 4 | 19 | 4 | 29 | 4 | 22 |  |
|  | ST27 | 17 | 4 | 19 | 4 | 29 | 4 | 22 |  |
|  | ST496 | 3 | 4 | 19 | 4 | 29 | 4 | 142 |  |
|  | ST227 | 3 | 4 | 19 | 4 | 29 | 22 | 22 |  |
|  | ST489 | 3 | 4 | 19 | 4 | 29 | 197 | 22 |  |
|  | ST787 | 3 | 4 | 19 | 4 | 29 | 48 | 22 |  |
|  | ST886 | 51 | 4 | 19 | 4 | 29 | 4 | 22 |  |
|  | ST435 | 3 | 4 | 31 | 4 | 29 | 4 | 22 |  |
|  | ST71 | 3 | 4 | 4 | 4 | 29 | 4 | 22 |  |
|  | ST72 | 3 | 4 | 4 | 4 | 29 | 4 | 22 |  |
| **CC345** |  |  |  |  |  |  |  |  |  |
|  | ST345 | 11 | 48 | 19 | 48 | 26 | 48 | 26 | ST189 and ST262 reflect a distant evolutionary relationship from CC345 |
|  | ST438 | 11 | 108 | 19 | 48 | 26 | 48 | 26 |  |
|  | ST88 | 11 | 48 | 43 | 48 | 26 | 48 | 26 |  |
|  | ST812 | 11 | 48 | 82 | 48 | 26 | 48 | 26 |  |
|  | ST189 | 11 | 48 | 3 | 48 | 26 | 48 | 26 |  |
|  | ST265 | 11 | 48 | 107 | 48 | 26 | 48 | 26 |  |
| **CC120** |  |  |  |  |  |  |  |  |  |
|  | ST120 | 60 | 108 | 86 | 98 | 18 | 45 | 51 | in one individual clusters |
|  | ST480 | 60 | 4 | 86 | 98 | 18 | 45 | 51 |  |
|  | ST188 | 60 | 108 | 86 | 99 | 18 | 45 | 51 |  |
|  | ST333 | 60 | 108 | 31 | 98 | 18 | 45 | 51 |  |
| **CC8** |  |  |  |  |  |  |  |  |  |
|  | ST8 | 28 | 4 | 82 | 88 | 63 | 69 | 1 | ST 341 has a distant evolutionary relationship from CC8 |
|  | ST482 | 28 | 4 | 82 | 4 | 63 | 69 | 1 |  |
|  | ST783 | 28 | 4 | 82 | 88 | 63 | 140 | 1 |  |
|  | ST341 | 28 | 4 | 127 | 88 | 63 | 69 | 1 |  |
| **CC332** |  |  |  |  |  |  |  |  |  |
|  | ST332 | 14 | 30 | 141 | 78 | 4 | 37 | 13 | ST328 has a distant evolutionary relationship from CC332 |
|  | ST789 | 14 | 30 | 141 | 78 | 4 | 35 | 13 |  |
|  |  |  |  |  |  |  |  |  |  |
|  | ST328 | 14 | 30 | 139 | 78 | 4 | 37 | 13 |  |
| **CC83** |  |  |  |  |  |  |  |  |  |
|  | ST83 | 5 | 52 | 27 | 13 | 17 | 25 | 40 | in one individual clusters |
|  | ST1 | 5 | 52 | 27 | 13 | 17 | 25 | 10 |  |
|  | ST264 | 5 | 52 | 27 | 132 | 17 | 25 | 40 |  |
|  | ST82 | 5 | 52 | 27 | 13 | 18 | 25 | 40 |  |
| **CC527** |  |  |  |  |  |  |  |  |  |
|  | ST527 | 43 | 41 | 107 | 42 | 37 | 40 | 33 | CC527 were divided into two branches, one contains ST69 and ST484, the other one contains ST527 and ST806 |
|  | ST806 | 244 | 41 | 107 | 42 | 37 | 40 | 33 |  |
|  | ST69 | 43 | 41 | 31 | 42 | 37 | 40 | 33 |  |
|  | ST484 | 43 | 41 | 19 | 42 | 37 | 40 | 33 |  |
| **CC890** |  |  |  |  |  |  |  |  |  |
|  | ST890 | 92 | 106 | 25 | 102 | 28 | 3 | 20 | in one individual clusters |
|  | ST134 | 92 | 116 | 25 | 102 | 28 | 3 | 20 |  |
|  | ST889 | 92 | 106 | 74 | 102 | 28 | 3 | 20 |  |
| **D1** |  |  |  |  |  |  |  |  |  |
|  | ST87 | 11 | 4 | 16 | 35 | 29 | 15 | 22 | grouped together |
|  | ST14 | 11 | 4 | 16 | 35 | 29 | 52 | 22 |  |
| **D2** |  |  |  |  |  |  |  |  |  |
|  | ST28 | 17 | 16 | 13 | 7 | 24 | 16 | 20 | grouped together |
|  | ST135 | 17 | 16 | 95 | 7 | 24 | 16 | 20 |  |
| **D3** |  |  |  |  |  |  |  |  |  |
|  | ST36 | 21 | 15 | 1 | 23 | 23 | 21 | 16 | grouped together |
|  | ST37 | 21 | 15 | 1 | 23 | 23 | 20 | 16 |  |
| **D4** |  |  |  |  |  |  |  |  |  |
|  | ST50 | 29 | 5 | 22 | 12 | 20 | 22 | 25 | grouped together |
|  | ST90 | 29 | 5 | 22 | 12 | 20 | 55 | 25 |  |
| **D5** |  |  |  |  |  |  |  |  |  |
|  | ST89 | 28 | 28 | 44 | 46 | 61 | 49 | 38 | grouped together |
|  | ST326 | 28 | 28 | 44 | 46 | 40 | 49 | 38 |  |
| **D6** |  |  |  |  |  |  |  |  |  |
|  | ST91 | 35 | 4 | 16 | 50 | 29 | 5 | 42 | grouped together |
|  | ST96 | 35 | 4 | 16 | 50 | 29 | 66 | 42 |  |
| **D7** |  |  |  |  |  |  |  |  |  |
|  | ST331 | 147 | 181 | 127 | 69 | 26 | 18 | 22 | grouped together |
|  | ST344 | 147 | 181 | 127 | 69 | 26 | 18 | 23 |  |
| **D8** |  |  |  |  |  |  |  |  |  |
|  | ST417 | 3 | 111 | 167 | 188 | 116 | 167 | 33 | grouped together |
|  | ST816 | 3 | 111 | 167 | 188 | 116 | 5 | 33 |  |
| **D9** |  |  |  |  |  |  |  |  |  |
|  | ST475 | 172 | 227 | 171 | 181 | 119 | 5 | 57 | grouped together |
|  | ST477 | 172 | 227 | 170 | 181 | 119 | 5 | 57 |  |
| **D10** |  |  |  |  |  |  |  |  |  |
|  | ST479 | 186 | 252 | 181 | 29 | 4 | 18 | 132 | grouped together |
|  | ST494 | 186 | 258 | 181 | 29 | 4 | 18 | 132 |  |
| **D11** |  |  |  |  |  |  |  |  |  |
|  | ST654 | 35 | 110 | 29 | 78 | 10 | 86 | 51 | grouped together |
|  | ST671 | 35 | 110 | 227 | 78 | 10 | 86 | 51 |  |

NOTE: Allele numbers of the housekeeping genes marked yellow represent the corresponding STs were occurred variation in these locus when compared with the ancestor of CCs or the other STs of Doublets. In this table, we speculate the difference between the results of eBRUST and ME tree was due to the variation of recA Gene.
